# Analysis of monocyte infiltration in MPTP mice reveals that microglial CX3CR1 protects against neurotoxic over-induction of monocyte-attracting CCL2 by astrocytes

**DOI:** 10.1186/s12974-017-0830-9

**Published:** 2017-03-21

**Authors:** Vincent R. Parillaud, Guillaume Lornet, Yann Monnet, Anne-Laure Privat, Andrei T. Haddad, Vanessa Brochard, Amaury Bekaert, Camille Baudesson de Chanville, Etienne C. Hirsch, Christophe Combadière, Stéphane Hunot, Christian S. Lobsiger

**Affiliations:** 1 Inserm, U 1127, F-75013 Paris, France; 2 CNRS, UMR 7225, F-75013 Paris, France; 3 Sorbonne Universités, UPMC Univ Paris 06, UMR S 1127, F-75013 Paris, France; 4 Institut du Cerveau et de la Moelle épinière, ICM, Hôpital Pitié-Salpêtrière, F-75013 Paris, France; 5 Present address: VIB Inflammation Research Center, Laboratory of Immunoregulation and Mucosal Immunology, Ghent University, Ghent, Belgium; 6 Sorbonne Universités, UPMC Univ Paris 06, Inserm U 1135, CNRS, ERL 8255, Centre d’Immunologie et des Maladies Infectieuses (CIMI-Paris), Paris, France

**Keywords:** Neuroinflammation, MPTP, Dopaminergic neurons, Chemokines, Monocytes, Astrocytes, Microglia, CCL2-CCR2, CX3CL1-CX3CR1, Parkinson’s disease

## Abstract

**Background:**

Evidence from mice suggests that brain infiltrating immune cells contribute to neurodegeneration, and we previously identified a deleterious lymphocyte infiltration in Parkinson’s disease mice. However, this remains controversial for monocytes, due to artifact-prone techniques used to distinguish them from microglia. Our aim was to reassess this open question, by taking advantage of the recent recognition that chemokine receptors CCR2 and CX3CR1 can differentiate between inflammatory monocytes and microglia, enabling to test whether CCR2^+^ monocytes infiltrate the brain during dopaminergic (DA) neurodegeneration and whether they contribute to neuronal death. This revealed unexpected insights into possible regulation of monocyte-attracting CCL2 induction.

**Methods:**

We used acute 1-methyl-4-phenyl-1,2,3,6-tetrahydropyridine **(**MPTP) mice and assessed monocyte infiltration by combining laser microdissection-guided chemokine RNA profiling of the substantia nigra (SN) with immunohistochemistry and CCR2-GFP reporter mice. To determine contribution to neuronal loss, we used CCR2-deletion and CCL2-overexpression, to reduce and increase CCR2^+^ monocyte infiltration, and CX3CR1-deletion to assess a potential implication in CCL2 regulation.

**Results:**

Nigral chemokine profiling revealed early CCL2/7/12-CCR2 axis induction, suggesting monocyte infiltration in MPTP mice. CCL2 protein showed early peak induction in nigral astrocytes, while CCR2-GFP mice revealed early but limited nigral monocyte infiltration. However, blocking infiltration by CCR2 deletion did not influence DA neuronal loss. In contrast, transgenic astrocytic CCL2 over-induction increased CCR2^+^ monocyte infiltration and DA neuronal loss in MPTP mice. Surprisingly, CCL2 over-induction was also detected in MPTP intoxicated CX3CR1-deleted mice, which are known to present increased DA neuronal loss. Importantly, CX3CR1/CCL2 double-deletion suggested that increased neurotoxicity was driven by astrocytic CCL2 over-induction.

**Conclusions:**

We show that CCR2^+^ monocytes infiltrate the affected CNS, but at the level observed in acute MPTP mice, this does not contribute to DA neuronal loss. In contrast, the underlying astrocytic CCL2 induction seemed to be tightly controled, as already moderate CCL2 over-induction led to increased neurotoxicity in MPTP mice, likely due to the increased CCR2^+^ monocyte infiltration. Importantly, we found evidence suggesting that during DA neurodegeneration, this control was mediated by microglial CX3CR1 signaling, which protects against such neurotoxic CCL2 over-induction by astrocytes, thus hinting at an endogenous mechanism to limit neurotoxic effects of the CCL2-CCR2 axis.

**Electronic supplementary material:**

The online version of this article (doi:10.1186/s12974-017-0830-9) contains supplementary material, which is available to authorized users.

## Background

Parkinson’s disease (PD) is characterized by dopaminergic (DA) denervation of the striatum and progressive death of DA neurons in the substantia nigra *pars compacta* (SNpc) [1]. Although there are familial forms, most are due to unknown primary causes. With patients diagnosed when the disease is already well underway, targeting the symptomatic phase is therefore therapeutically relevant.

Neuroinflammatory processes are present in most neurodegenerative disorders, including PD, and while not considered primary causes, they could contribute to the symptomatic phase [2]. During CNS neurodegeneration, neuronal damage leads to activation of microglia and astrocytes, which in turn can amplify the neuroinflammatory response by chemokine secretion leading to CNS infiltration of peripheral immune cells. In human PD postmortem SN, there are still activated microglia and infiltrated lymphocytes present [2, 3]. Studies in the acute neurotoxic 1-methyl-4-phenyl-1,2,3,6-tetrahydropyridine (MPTP) mouse model provided evidence that neuroinflammatory processes can indeed contribute to nigral DA neuronal death. This included genetic deletions of microglial effectors [4] or suppression of T lymphocytes [3], which both reduced neuronal loss, suggesting that neuroinflammation in PD actively participates to neuronal death.

Although presence and role of brain infiltrating peripheral lymphocytes have been well analyzed in mouse models of DA neurodegeneration, the situation for peripheral monocytes is much less clear and remains highly controversial. Essentially, this is because of the technical challenge to distinguish microglia (the CNS resident macrophages) from infiltrating blood monocytes becoming tissue macrophages [5, 6]. Of note, CNS infiltrating lymphocytes are much easier to detect, due to well-established antibodies working in tissue-sections [3], which are lacking for monocytes. The classic method to determine CNS infiltration of blood monocytes consists of irradiation followed by bone marrow transplantation (BMT) using donor green fluorescent protein (GFP) mice. However, it has been demonstrated that this strategy leads, through blood-brain-barrier damage and flooding of the circulation with hematopoietic stem cells, to artifact brain infiltration of peripheral immune cells (including monocytes), both in the baseline and especially during neurodegenerative conditions [7–9]. While several previous studies have assessed the presence of CNS infiltrating monocytes in the MPTP model of DA neurodegeneration and reported significant infiltration, they all used this artifact-prone technique [10–13]. In addition, none of them assessed whether blocking such CNS monocyte infiltration would affect death of DA neurons in the SNpc. Thus, they remain silent on actual presence and role of brain infiltrating peripheral monocytes during DA neurodegeneration.

A recent reassessment of the cellular expression of chemokine receptors on microglia and blood monocytes [14–16] has now provided new tools to readdress this open question. Chemokines are a large family of around 40 ligands and 20 receptors forming defined ligand-receptor axes and are implicated in attraction of immune cells to inflamed tissues [17, 18]. Inflammatory-type (classical) blood monocytes are known to express the chemokine receptor CCR2, which is required for their infiltration into inflamed peripheral tissues that express (in mice) one or several of the three chemokines CCL2/7/12 [19]. Microglia strongly express the chemokine receptor CX3CR1 (the fraktalkine receptor), which is important for homeostatic interactions with CX3CL1/fraktalkine expressing neurons [17]. Of note, CX3CR1 is in mice also expressed by the (non-classical) patrolling-type CCR2^−^ monocyte subpopulation, but they are less prominent than CCR2^+^ inflammatory-type monocytes and not the principal monocyte cell type implicated in inflammation-linked tissue infiltration [20]. In part due to lack of CCR2 antibodies working in tissue sections, it has been unclear whether microglia express CCR2. Recently developed fluorescent reporter mice for CCR2 and CX3CR1 have now established that CCR2 is *not* expressed by resting or activated microglia, while CCR2^+^ monocytes express only very low levels of CX3CR1 [16]. Although CCR2 will get downregulated once CCR2^+^ monocytes have infiltrated the tissue (and became macrophages) [19], a window of CCR2 expression should remain [6], especially considering the relative stability of cytoplasmic expressed GFP. Thus, CCR2 reporter mice provide a new tool to reassess brain infiltration of inflammatory monocytes without the need for artifact-prone irradiation/BMT strategies.

Of note, several previous studies have used CCL2 or CCR2 deletion to assess their role in the MPTP model and suggested no effect on DA neurodegeneration [21–23]. However, at that time, none of them made a link to brain infiltrating monocytes but rather suggested the CCL2-CCR2 axis to be implicated in brain resident reactive responses, with the actual cell types involved remaining elusive. Importantly, the only study that used deletion of the crucial monocyte receptor CCR2 [23], only reported on DA denervation in the striatum but not on actual death of DA neurons in the SNpc, using a MPTP regiment too low to induce actual loss of DA neuronal. The other two studies only assessed deletion of the ligand CCL2 [21, 22], but the effects could easily be masked by compensation through the co-induced CCL7/12 ligands.

Thus, despite significant previous efforts, surprisingly, the actual questions whether inflammatory CCR2^+^ monocytes do infiltrate the brain and whether they contribute to loss of nigral DA neurons in a model of DA neurodegeneration, remain unanswered. With the present study, we used the acute MPTP model of DA neurodegeneration [24] and applied two direct methods to provide answers to these two long-standing open questions—transgenic CCR2-GFP reporter mice to track brain infiltration of inflammatory CCR2^+^ monocytes and CCR2 deletion to block such potential infiltration and assess its role. This revealed early but limited infiltration of CCR2^+^ monocytes into the affected SNpc in MPTP mice, but no direct contribution to death of DA neurons. Complementary to the analysis of CCR2^+^ monocyte infiltration, we also determined the cellular source and regulation of the underlying induction of the corresponding CCL2/7/12 chemokine ligands. This revealed that astrocytes are the main source for induction of monocyte-attracting chemokines in MPTP mice. Importantly, by analyzing CCL2 regulation, we found evidence suggesting that microglial CX3CR1-signaling controls astrocytic CCL2 induction. This could protect against astrocytic CCL2 over-induction, which would otherwise become neurotoxic and aggravate DA neurodegeneration.

## Methods

### Animals

Mice were kept under a 12-h light/dark cycle and had ad libitum access to food and water. Animal handling was carried out according to ethical guidelines and experimental procedures approved by the French MESR Ministery (protocol no. #02514.01 to C.S.L.). *Mouse lines used*: C57BL/6J (Janvier, France and #000664, JAX, USA), CCR2-GFP (heterozygous BAC-transgenic mice, expressing GFP from the mouse CCR2 locus, without expression of transgenic *Ccr2*; a kind gift of Dr. Eric G. Pamer, Sloan-Kettering Institute, New York, USA) [25], CCR2-KO (#004999, JAX, USA; homozygous knock-out mice deleted for *Ccr2*), GFAP-CCL2 (#014095, JAX, USA; heterozygous transgenic mice, expressing mouse *Ccl2* from the human *GFAP* promotor), CX3CR1-GFP (#008451, JAX, USA; homozygous knock-in mice deleted for *Cx3cr1* by introduction of *GFP*); CX3CR1-KO (homozygous knock-out mice deleted for *Cx3cr1*), CCL2-KO (#004434, JAX, USA; homozygous knock-out mice deleted for *Ccl2*). *Of note*, all the lines were on a C57BL/6J genetic background, important when using the MPTP model [24] and showed robust 30% loss of DA neurons at 7 days after acute MPTP intoxication (see “Results” section). *Genotyping*: all primers and conditions were used according to JAX (USA) or for CCR2-GFP as previously described [25]. Of note, GFAP-CCL2 mice can develop a neurological phenotype at advanced ages (10–12 months old) due to CNS leukocyte infiltration [26]. However, at the young ages used for our studies (3–4 months old), their CNS shows no signs of pathological neuroinflammation [26].

### MPTP model

Upon arrival, mice were acclimated for at least 1 week before MPTP treatment and kept for the duration of the treatment in a temperature-controlled (22 °C) and ventilated cabinet. For all experiments, the acute MPTP paradigm was used [24]. Mice received 4 intraperitoneal (i.p.) injections of either a control saline solution or MPTP-HCl (20 mg/kg; Sigma) at 2-h intervals and were kept for 48 h at 28 °C before returning to 22 °C for the rest of the experiment (up to 14 days). Depending on the mouse line, MPTP cumulative doses resulted in mortality rates between 10–30%. All MPTP experiments were done with 12–16-week-old male mice.

### Laser microdissection

The protocol was adapted from [27]. Deeply anesthetized mice (by i.p. injection of a mixture of ketamine [Virbac/France] at 100 mg/kg and xylazine [Bayer/France] at 10 mg/kg in 0.9% NaCl) were rapidly perfused with 25 ml of ice-cold saline (to remove blood from the brain) and sacrificed by spinal dislocation. The brains were rapidly dissected, frozen in isopentane (−30 °C) and stored at −80 °C. Fifteen-microliter brain sections were collected at −20 °C on RNAse-free PEN-membrane slides (Leica) using a cryostat. Slides were immediately frozen in a box with desiccate on dry ice, stored at −80 °C and used within a week. The region of the SNpc containing the highest density of DA neurons was fully collected as *consecutive* sections. Identification of the SNpc region containing the highest density of DA neurons was done by an adapted rapid Nissl stain to prevent RNA degradation. Nissl staining for laser microdissection (LMD) was as follows: removal of PEN-slides from −80 °C, rapid drying of slides using a 1200 W hairdryer (30 s), EtOH 50% (30 s), EtOH 75% (1 min), EtOH 95% (10 s), EtOH 100% (15 s), drying (with hairdryer for 15 s), 4% cresyl-violet-acetate (in water with 0.3% glacial acetic acid) (30 s), 2× H_2_O (5 s each), EtOH 70% (30 s), EtOH 95% (10 s), 2× EtOH 100% (30 s), and final drying (with hairdryer for 30 s). Slides were further dried in a dessicator for 1–5 h before LMD. To isolate the SNpc subregion with the highest density of DA neurons, LMD was performed at a ×5 magnification using both a Zeiss PALM Microbeam and a Leica LMD-7000 system. A total of 50 SNpcs were microdissected per mouse/sample, and SNpcs were collected into dry RNAse-free 0.5 ml adhesive tube caps (Zeiss). Twenty milliliters of RLT buffer (with 1% β-mercaptoethanol, RNeasy Microprep Kit; Qiagen) were added to the tube cap followed by homogenization (vortex and pipetting), freezing on dry ice and storing at −80 °C.

### RNA extraction

For all LMD samples, RNA was extracted with the RNeasy Microprep Kit (Qiagen) with DNase treatment and eluted in 12 μl of RNAse-free water. RNA integrity (RIN value) and RNA concentrations were assessed on a Bio-Analyzer (Agilent Technologies) using the Pico Assay II Chips according to manufacturer’s instructions and on a NanoDrop (Thermo Fisher). For 50 collected SNpcs, we obtained around 50 ng of total RNA with a RIN >7.

### TaqMan microfluidic card RT-qPCR

To analyze chemokine expression in laser-microdissected SNpc regions, we first produced single-stranded cDNA (High-Capacity cDNA Reverse Transcription Kit/wRNase Inhibitor; Applied Biosystems) from 50 ng of total RNA (coming from 50 microdissected SNpc per mouse/sample) using random hexamers, according to the manufacturer’s instructions in 20 μl reaction volumes. For reverse transcription (RT), all 36 samples (1 mouse/sample; with 9 control samples and 27 MPTP samples, consisting of 9 MPTP samples for the 2, 4, and 7 day timepoints) were processed in parallel in a 96 multiwell plate on a GeneAmp PCR System 9700 (Applied Biosystems) with the following conditions: 25 °C (10 min), 37 °C (120 min), and 85 °C (5 s) followed by cooling to 4 °C. Due to the limited amount of RNA, linear PCR pre-amplification was used, followed by TaqMan qPCR on 384-well microfluidic cards. Pre-amplification was performed with 7.5 μl of the resulting 20 μl of cDNA using the TaqMan PreAmp Master Mix (Applied Biosystems) with a custom-made TaqMan PreAmp Assay Pool (that contained the TaqMan assays for all the 96 genes from the custom-made TaqMan qPCR microfluidic cards) according to the manufacturer’s instructions in a final reaction volume of 50 μl. Pre-amplification was performed on a GeneAmp PCR System 9700 (Applied Biosystems) using the following conditions: 95 °C (10 min), 14 cycles—95 °C (15 s), 60 °C (4 min). Reactions were stored at −20 °C for a maximum of 1 week. Custom-made TaqMan qPCR 384-well microfluidic cards (Applied Biosystems) were designed with the 4 × 96 assay format, containing 96 individual genes (for singlet analysis), allowing the processing of 4 samples per array. A total of 9 cards were used for the 36 samples. Primer/probe sets were chosen from the Applied Biosystems repertoire and whenever possible to span an intron. Care was taken to choose primer/probe sets to amplify from the middle of the coding sequence and to amplify only small amplicons (60–100 bp). For the qPCR reactions, 1/5 of the pre-amplified cDNA was used per mouse/sample, and qPCR reactions were performed using the TaqMan Gene Expression Master Mix (Applied Biosystems) according to the manufacturer’s instructions in a final reaction volume of 100 μl. This was distributed over 96 wells (4 rows of the 384-well microfluidic card) containing the 96 genes, resulting in a final reaction volume of 1 μl per micro-well. The qPCR reaction was performed on the 7900HT Fast Real-time PCR System (Applied Biosystems) coupled to a robotic plate loader (Applied Biosystems) with the following conditions: 50 °C (2 min), 94.5 °C (10 min), 40 cycles—97 °C (30 s), and 59.7 °C (1 min).

### Analysis of TaqMan microfluidic card qPCR

We compared the three different MPTP groups (2, 4, and 7 days after intoxication; *n* = 9 mice per group, as biological replicates) to the saline samples (*n* = 9 mice) and identified statistical significant fold changes (*P* < 0.05; ANOVA with Holm-Sidak post hoc analysis). Data analysis was performed with SDS RQ Manager 2.3c/DataAssist 2.0 (Applied Biosystems) and qBasePlus (Biogazelle). For normalization, two reference genes (*Gapdh* and *Hprt1)* were assessed, and *Hprt1* was chosen as it did show the least variation among all the 36 samples (gNORM; Biogazelle, Ghent, Belgium). In addition, we confirmed with one sample that did not undergo an RT reaction that none of the 96 primer/probe sets amplified potential genomic contaminations. Pre-amplification and qPCR for one SNpc sample showed that 88/96 genes amplified with Ct values <30 (as an upper limit for a positive signal) indicating successful pre-amplification.

### Immunohistochemistry

Deeply anesthetized mice were perfused with 25 ml of saline (with 5 U/ml of heparin), followed by 100 ml of 4% paraformaldehyde (PFA) to fix the brain tissue. The brains were dissected out and postfixed at 4 °C for 24 h in 4% PFA followed by 48 h cryoprotection in 30% sucrose (in PBS), frozen in isopentane (−30 °C) and stored at −80 °C. Perfused brains were sectioned at 20 μm on a freezing microtome (Leica) (at the midbrain level to collect the SNpc) and sections stored in PBS (4 °C). Floating sections were rinsed three times with PBS 0.1 M (5 min) and blocked for 45 min in PBS, 4% BSA (Sigma), 0.3% Triton X100 (Sigma). Primary antibodies were incubated in PBS/0.1% Triton X100 for 48 h at 4 °C (under mild agitation) and washed in PBS. For anti-chemokine antibodies, PBS/0.1% Triton X100 was used for all washing steps. For the anti-collagen IV antibody, demasking was used (4 min at 37 °C) in proteinase-K (10 μg/ml; Sigma) and 0.5% Triton X100. For immunofluorescence stainings, secondary antibodies were used for 1 h at room temperature in PBS, followed by washing in PBS. Sections were mounted on gelatinized slides, dried, and cover-slipped using Fluoromount-G (Southern Biotech). For immunohistochemical stainings, before blocking, endogenous peroxidases were inhibited by incubating sections for 5 min in 0.1 M PBS containing 20% MetOH and 0.9% H_2_O_2_. Biotinylated secondary antibodies (1/250; rabbit anti-goat IgG and goat anti-rabbit IgG; Vector Labs) were used for 1 h at room temperature in PBS. After washing (PBS), sections were incubated for 1 h in Elite Vectastain ABC amplification solution (1/150; Vector Labs) followed by washing in PBS and TB 0.25 M, and revealed with 3,3′-diaminobenzidine (DAB; VectorLabs). Sections were mounted on gelatinized slides, dried, and dehydrated: 2× H_2_O (30 s), EtOH 70% (30 s), EtOH 95% (30 s), 2× EtOH 100% (30 s) and 2× Xylene (2 min), then cover-slipped with Permount (Sigma). *Primary antibodies*: rabbit polyclonal antibodies against Iba-1 (1/500; Wako), TH (1/500; US Biological), GFAP (1/3000; Dako), collagen IV (1/400; Abcam) and GFP (1/500; Invitrogen); goat polyclonal against CCL2 (1/200; R&D), CCL12 (1/200; R&D), CXCL16 (1/100; R&D) and CCL7 (1/200; R&D); mouse monoclonal against TH (1/500; Immunostar); rat monoclonal against CD11b (1/250; Serotec), and chicken polyclonal against GFP (1/500; Invitrogen) were used. *Secondary antibodies*: highly cross-absorbed Alexa 488 (1/500; Invitrogen; donkey anti-rabbit, donkey anti-mouse, goat anti-chicken), Alexa 555 (1/500; donkey anti-goat, donkey anti-rabbit, goat anti-rat) and Alexa 647 (1/500; donkey anti-mouse) secondaries were used. *Lectin*: DyLight 594 Lectin (1/500; VectorLabs) was used for 1 h at room temperature in PBS.

### Image analysis and cellular quantification

All fluorescent stainings were analyzed on a Zeiss AxioImager microscope using FluoUp software (Explora Nova, France) and Photoshop CS6 (Adobe, USA). Conditions for tissue processing, immunostaining, and image capturing were kept constant for all animals. Quantification of MPTP-induced loss of DA neurons in the SNpc: DAB-immunostained sections were analyzed by bright-field microscopy, using a Leica DM4000 semi-automated microscope equipped with image analysis software (Mercator; Explora Nova, France). TH-positive DA neurons were quantified stereologically on ten regularly spaced 20-μm thick sections (every 10th) covering the whole SNpc (six of these ten sections contain clear SNpc regions) using the VisioScan stereology tool [3, 28]. Quantification of CCR2-GFP^+^ cells and of CCL2^+^ cells in the MPTP affected SNpc was done similarly (DAB-immunostainings and quantified stereologically). Briefly, to estimate the number of CCR2-GFP^+^ cells in the entire SNpc at the peak of infiltration (36 h after MPTP intoxication): CCR2-GFP^+^ cells were mainly detected in the rostral-medial part of the SNpc (on four of six clear SNpc sections). If in a MPTP treated mouse, we would count ten CCR2-GFP^+^ cells on a single SNpc section (bilateral), this would result in: 10 × 4 × 10 = 400 cells per full SNpc. A similar strategy was used to count CCL2^+^ and CCR2-GFP^+^ cells in the striatum. For all quantifications, the investigator was blinded to the treatment and genotype groups during the analysis.

### Stereotactic LPS injection

C57BL/6J (*n* = 3) and CCL2-KO (*n* = 3) mice (males, 3 months old) were anesthetized with ketamine (66 mg/kg) and xylazine (6.6 mg/kg) in 0.9% NaCl (i.p.). Mice were placed on a digital stereotaxic frame (David Kopf Instruments, USA) and injected with a stainless canula (10 μl syringe, #1701, 26G; Hamilton, Switzerland) into the SN (medio-lateral +1.3 mm, antero-posterior −2.9 mm and dorso-ventral −4,6 mm from the bregma). One microgram of sterile *Escherichia coli* LPS (Sigma L4391; serotype, E.C. 0111:B4) was unilateraly injected into the SN (0.2 μl/min, 1 μl injected). After injection, the needle was left for 5 min and removed slowly. After recovery (2 h/37 °C) kept under standard conditions for 48 h until perfusion.

### Statistical analysis

All values were expressed as the mean ± SEM. Differences in means between two groups were analyzed using one-way ANOVA followed by All Pairwise Multiple Comparison Procedures (Holm-Sidak method), or when data were not normally distributed, with a Dunn test. Differences in means among multiple data sets were analyzed using one- or two-way ANOVA with time, treatment, or genotype as the independent factors. When ANOVA showed significant differences, pairwise comparisons between means were tested by Holm-Sidak post hoc analysis. When data were not normally distributed, a Kruskal-Wallis ANOVA on rank test was used, followed by pairwise comparison using the Dunn test. In all analyses, *P* < 0.05 was considered significant (SigmaStat 4.0, SigmaPlot 11; Systat Software, CA, USA).

## Results

### Chemokine RNA profiling in the substantia nigra of MPTP mice reveals early CCL2/7/12-CCR2 axis induction

**Fig. 1:**
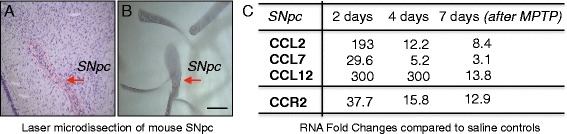
Chemokine RNA profiling in the substantia nigra of MPTP mice reveals early CCL2/7/12-CCR2 axis induction. **a**–**b** Laser microdissection (LMD) of Nissl-stained substantia nigra *pars compacta* (SNpc) from mouse midbrain tissue sections, used for TaqMan RT-qPCR profiling of the full chemokine family in MPTP mice. **a** Showing a SNpc before LMD and **b** showing multiple SNpcs collected by LMD. (*Scale bar*; **b**, 200 μm). **c** Selected results, showing fold change (*P* < 0.05; ANOVA with Holm-Sidak test) inductions (RNA) of the CCL2/7/12-CCR2 (ligand receptor) axis at 2, 4, and 7 days (2d/4d/7d) after acute MPTP intoxication, suggesting nigral CCR2^+^ monocyte infiltration. Data are shown as average fold changes compared to saline controls; *n* = 9 mice per condition, normalized to *Hprt1* (for a full list of results, see Additional file 2: Table S1)

As brain infiltration of peripheral immune cells during neurodegeneration is likely controlled by local induction of chemokines in the affected brain regions, we first performed global RNA profiling of the full chemokine ligand-receptor family within the microdissected SNpc during the course of DA neurodegeneration in MPTP mice. This approach allowed us not just to identify the chemokine ligand-receptor axes indicative of monocyte infiltration but also to compare the kinetics of such potential monocyte infiltration with the known infiltration of lymphocytes. We chose the MPTP mouse model and used an acute intoxication paradigm [24], as this model has been extensively used to study contribution of neuroinflammatory processes to DA neurodegeneration [4, 29, 30], including the deleterious contribution of infiltrating T lymphocytes [3]. Chemokine ligands should be induced by local glial cells, with the corresponding receptors expressed by peripheral lymphocytes and monocytes [17]. Due to lack of good tissue antibodies for many mouse chemokines, we used RNA profiling with highly specific TaqMan qPCR arrays (for all 37 mouse chemokines and 24 receptors) [18] and applied laser microdissection (LMD) [27] to isolate only the SNpc, avoiding dilution effects from the less affected neighboring ventral tegmental area [28] (Fig. 1a, b). Analysis was done at 2, 4, and 7 days after MPTP intoxication, corresponding to *before*, *during*, and *after* the peak of DA neuronal loss, respectively [3, 31]. This model shows early microglial activation (at 1–2 days), followed by astrogliosis (at 2–4 days) and leads to stable death of around 30% of DA neurons at 7 days (Additional file 1: Figure S1) [3, 32]. In addition, infiltrating lymphocytes are present in the affected SNpc already at 2 days [3]. To correlate chemokine profiles with the overall neuroinflammatory process, we also assessed additional markers, including for microgliosis, astrogliosis, and lymphocyte presence.

Results confirmed persistent downregulation of *Th* from affected DA neurons in the SNpc and induction of an early neuroinflammatory response that persisted throughout the neurodegenerative process (microgliosis: *cd68*, *cd11c*, *MHC-II*, *B2m*, *Cx3cr1*; astrogliosis: *Gfap*; lymphocytes: *cd3c*, *Tbx21*) (Additional file 2: Table S1A, C). From the 37 tested chemokines, 27 showed significant regulations during DA neurodegeneration, while ten were not expressed or not regulated (Additional file 2: Table S1B). We could separate the chemokines into four different temporal expression profiles (Additional file 3: Figure S2 and Additional file 2: Table S1B). (*Profile A*): ten chemokines showing strong early peak induction at 2 days, then less strong induction at 4 and 7 days (*Ccl2*/*3*/*4*/*7*/*8*/*12*/*22*, *Cxcl2*/*10*/*11*); (*Profile B*): ten chemokines showing early but persistent induction (at 2/4 and 7 days) (*Ccl5/9/11/19*, *Cxcl1/5-6/4/9/14/16*); (*Profile C*): two chemokines showing increased upregulation at the late timepoint (*Ccl6*, *xCl1*), and (*Profile D*): four chemokines showing downregulation (*Ccl20/28*, *Ccl17*/*Cxcl13*). In addition, one chemokine (CCL24) showed a mixed regulation (early down, then upregulated). Interestingly, chemokines with early peak induction (*Profile A*), seemed to be almost absent under control conditions (Additional file 2: Table S1B). Importantly, for most of the early induced chemokines (16/20; except for *Ccl11*/*19*/*22* and *Cxcl14*), the corresponding receptors were expressed and likewise early induced, suggesting induction of functional chemokine axes (Additional file 2: Table S1B).

As our primary focus was whether there is nigral infiltration of monocytes during DA neurodegeneration, we were most interested in the CCL2/7/12-CCR2 (ligand receptor) axis (Fig. 1c). The major cell type expressing CCR2 are inflammatory blood monocytes, which are attracted to inflamed tissues by CCL2/7/12, of which CCL2 (MCP-1) is the best described one [19]. Importantly, our RNA results showed both early induction (at 2 days) of CCL2/7/12 and parallel to it, early induction (at 2 days) of CCR2. Regarding the peripheral immune cell infiltration, our RNA screen also revealed strong and early induction of chemokine axes that could be important for the known deleterious infiltration [3] of peripheral T lymphocytes (CCL3/4/5-CCR1/5, CXCL10-CXCR3, and CXCL16-CXCR6) (Additional file 3: Figure S2B and Additional file 2: Table S1B). Interestingly, when comparing the kinetics of the different chemokine axes, the monocyte (CCL2/7/12-CCR2) axis showed a rather early peak induction, while the T lymphocytes axes showed a more maintained induction (Additional file 3: Figure S2B and Additional file 2: Table S1B, C).

Thus, our RNA profiling shows early induction of the monocyte-linked CCL2/7/12-CCR2 axis in the affected SNpc of MPTP mice.

### Astrocytes are the main source for early induction of monocyte-attracting chemokines CCL2 and CCL7 in the affected substantia nigra of MPTP mice

Next, we confirmed RNA induction of CCL2/7/12 on a protein level and determined their precise cellular source in the MPTP affected SNpc. For our initial RNA screen, we had to focus on a limited number of timepoints and chose as an overview, 2/4 and 7 days after MPTP intoxication. However, since the chemokines, we were most interested in, showed a strong induction already at 2 days (*Profile A*; CCL2/7/12, Fig. 1c and Additional file 3: Figure S2B), it was possible that the real peak induction happened even earlier (before 2 days). Thus, to determine protein induction, we expanded our initial timecourse with three earlier timepoints (12, 24, and 36 h).

**Fig. 2:**
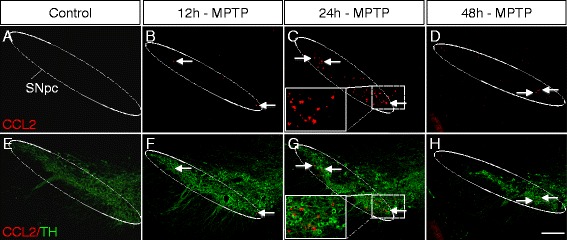
Early but transient CCL2 protein induction within the affected substantia nigra of MPTP mice. **a**–**h** Immunofluorescence stainings showing the timecourse of CCL2 protein induction in the SNpc (*dotted white line*) of MPTP mice, at 12 h **(b**, **f)**, 24 h **(c**, **g)**, and 48 h/2 days **(d**, **h)** after acute MPTP intoxication, compared to saline injected controls **(a**, **e)**. CCL2 in *red* and TH (marking DA neurons) in *green*. While CCL2 is absent in controls **(a**, **e)**, first signs of CCL2 induction appear at 12 h **(b**, **f)** (*arrows*), followed by robust induction at 24 h **(c**, **g)** (*arrows* and *insets*) and reduced induction at 36 h *(data not shown)* and 48 h/2 days **(d**, **h)** (*arrows*). CCL2 induction does not colocalize with neuronal TH staining (*insets* in **c** and **g**). No CCL2 induction was detected at 4 and 7 days *(data not shown)*. (*Scale bar*; **h**, 200 μm)

**Fig. 3:**
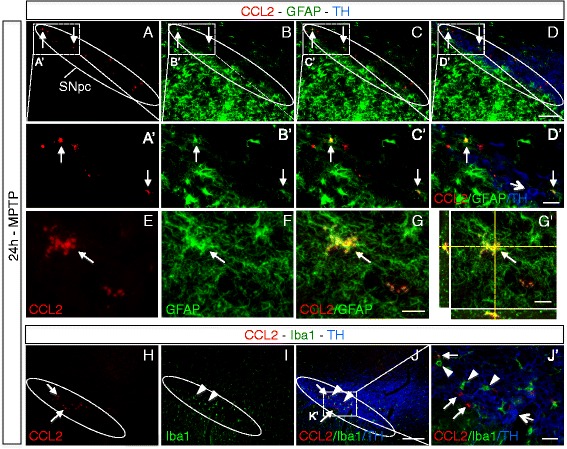
Astrocytes are the main source for early CCL2 induction within the affected substantia nigra of MPTP mice. **a**–**g** Immunofluorescence stainings showing colocalization of *CCL2* (*red*, *arrows*) with *GFAP* (*green*, *arrows*) positive astrocytes (but not *TH* positive DA neurons; *blue*, *open arrow* in **D**’) within the *SNpc* of MPTP mice at 24 h after intoxication (the peak of CCL2 induction, see Fig. 2). **a**–**d**) Overview images of several *CCL2*/*GFAP* double-positive cells (*arrows*) (with enlargements in **a**’–**d**’). **e**–**g** Confocal images of *CCL2*/*GFAP* colocalization, with an orthogonal view in **(g**’**). h**–**j)** No colocalization of *CCL2* (*red*, *arrows*) with Iba1 (*green*, *arrowheads*) positive microglial cells (or *TH* positive DA neurons; *blue*, see *open arrow* in enlargement, **j**’) within the *SNpc* of MPTP mice. (*Scale bars*; **d** and **j** *,* 200 μm; **d**’ and **j**’, 40 μm; **g**, 20 μm)

Interestingly, we detected induction of CCL2 protein expression exclusively in the affected SNpc, already at 12 h after MPTP intoxication, which reached a clear peak at 24 h, followed by less strong expressions at 36 h and 2 days with no signal at 4 and 7 days (Fig. 2). Importantly, confocal analysis revealed that the CCL2 expressing cells were not (Iba1^+^) microglia but (GFAP^+^) astrocytes (Fig. 3). Of note, we verified the specificity of the anti-CCL2 antibodies we used, with CCL2 deleted mice (Additional file 4: Figure S3). In parallel to CCL2, we also assessed protein induction of the two other CCR2 ligands, CCL7 and CCL12 (Additional file 5: Figure S4). As for CCL2, we detected CCL7/12 inductions, with a peak at 24 h, exclusively in cells of the affected SNpc. Interestingly, while CCL7 was astrocytic and not expressed by microglia, CCL12 (a mouse specific chemokine) was only induced in microglia (Additional file 5: Figure S4). Of note, all three chemokines showed comparable vesicular stainings in MPTP mice, while saline injected control mice were completely blank (Fig. 2 and Additional file 5: Figure S4), consistent with the RNA data that showed strong induction, over almost absent signals in controls (Additional file 2: Table S1B). A further indication for the specificity of the vesicular CCL2/7/12 stainings, was our observation that an unrelated chemokine we tested for another study, CXCL16, that represents beside CX3CL1 the only membrane-bound chemokine, showed not a vesicular but a rather membranous staining (Additional file 6: Figure S5). In the SNpc, we only detected intracellular CCL2/7/12 stains, although extracellular or vessel-associated stains could be possible. However, such stains are likely to be very weak and risk to be washed out due to the initial perfusion.

Thus, on a protein level, we found strong early but transient induction of CCL2/7 in the affected SNpc, and we revealed that the major source for these CCR2^+^ monocyte-attracting chemokines were astrocytes.

### Transgenic CCR2-GFP reporter mice reveal early but limited infiltration of CCR2^+^ monocytes into the affected substantia nigra of MPTP mice

So far, our results showed messenger RNA (mRNA) induction of the CCL2/7/12-CCR2 axis in the affected SNpc and on a protein level, astrocytic CCL2/7 induction. We next sought to determine the presence of nigral infiltration of CCR2^+^ monocytes, a controversial question that remained open (see “Background”). To avoid artifact-prone irradiation/BMT strategies [7, 8], we took advantage of the recently generated BAC-transgenic CCR2-GFP reporter mice [25]. These mice have been well established to mark all blood CCR2^+^/Ly6C^high^ inflammatory monocytes uniformly with GFP [25].

**Fig. 4:**
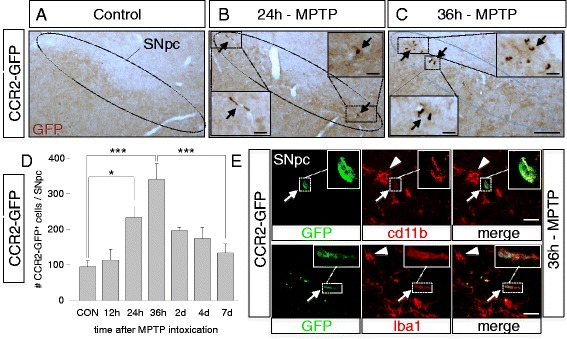
CCR2-GFP reporter mice reveal early but limited nigral infiltration of CCR2^+^ monocytes during MPTP mediated DA neurodegeneration. **a**–**c** Timecourse analysis of appearance of CCR2-GFP^+^ cells (stained with anti-*GFP* antibodies, *brown*, *arrows*), at 24 h (**b**) and 36 h (**c**) after acute MPTP intoxication within the *SNpc* of CCR2-GFP mice and compared to saline injected controls (**a**). Compared to only very rare CCR2^+^ cells detectable in controls, multiple CCR2-GFP^+^ cells are detected at 36 h, (see magnification *insets* in **b** and **c**). **d** Quantification of CCR2-GFP^+^ cells within the *SNpc* at 12 h (*n* = 3), 24 h (*n* = 5), 36 h (*n* = 4), 48 h/2 days (*n* = 5), 4 days (*n* = 4), and 7 days (*n* = 6) after MPTP intoxication in CCR2-GFP mice (compared to saline injected controls; CON, *n* = 3), suggesting early but limited CCR2^+^ monocyte infiltration, with a peak at 36 h, then returning to baseline levels after 4/7 days. Counts represent the estimated total of CCR2-GFP^+^ cells within the entire *SNpc* (means +/− SEM; *n* = 3–6 mice per condition; *P = 0.048, ***P < 0.001; Kruskal-Wallis test). **e** Immunofluorescence stains in the *SNpc* of CCR2-GFP mice at 36 h after MPTP intoxication, showing colocalization of CCR2-GFP^+^ cells with myeloid markers *CD11b* and *Iba1* (*insets* for magnification), beside resident *GFP* negative microglia (*arrowhead*) (*Scale bars*; ***C***, 200 μm, with 10 μm in *insets*; ***E***, 10 μm)

Thus, we performed a full timecourse analysis to assess CCR2^+^ monocyte infiltration into the SNpc of CCR2-GFP mice, at 12, 24, 36 h, and 2, 4, and 7 days after MPTP intoxication (Fig. 4a–d). Of note, our initial RNA screen was done at 2/4 and 7 days and showed a strong CCR2 induction already at 2 days (Fig. 1c). As above, for the protein induction of the corresponding ligand CCL2, actual peak CCR2^+^ monocyte infiltration could happen earlier than at 2 days—thus, our choice to include three earlier timepoints (12, 24, and 36 h).

The brains of control CCR2-GFP mice contained only rare CCR2-GFP^+^ cells mainly in/around vessels and some associated with meninges (*data not shown*). We confirmed robust DA neuronal loss at 7 days after acute MPTP intoxication for this CCR2-GFP reporter line (28% compared to saline controls, *P* < 0.05, Holm-Sidak method; actual counts of DA neurons (as means +/− SEM) were: 12,040 +/− 302 for saline, *n* = 3 mice; 8680 +/− 278, for MPTP, *n* = 10). Interestingly, compared to saline injected CCR2-GFP mice, we observed more frequent presence of CCR2-GFP^+^ cells within the affected SNpc of MPTP mice, already at 24 h, followed by a peak at 36 h, after which numbers rapidly declined at 48 h and reached baseline levels at 4 and 7 days (Fig. 4a–d). The morphology of these CCR2-GFP^+^ cells was rather roundish/elongated (Fig. 4b–c and e). Of note, in a recent study analyzing CNS monocyte infiltration in the multiple sclerosis EAE model and using similar CCR2 reporter mice, the same roundish/elongated morphology of infiltrating CCR2^+^ monocytes was detected [6]. We detected CCR2-GFP^+^ cells, both closely associated with blood vessels but also within the parenchyma (Additional file 7: Figure S6). Although at 36 h, CCR2-GFP^+^ cells were clearly visible in the MPTP-affected SNpc (Fig. 4c), their estimated total number within the full SNpc remained however low, reaching 340 cells (see also “Methods”) (Fig. 4d). Importantly, all of the CCR2-GFP^+^ cells within the affected SNpc double-stained for CD11b or Iba1 (classic markers present on monocytes/macrophages and microglia) (Fig. 4e), strongly suggesting that they are of myeloid origin and thus infiltrating CCR2^+^ monocytes. However, CCR2-GFP^+^ cells expressed less CD11b or Iba1 than neighboring microglia (Fig. 4e). A possible explanation could be that infiltrating CCR2^+^ monocytes express lower levels of these markers than fully differentiated tissue macrophages.

In addition to the SNpc containing the DA neuronal cell bodies, we also analyzed their main target, the striatum. While we detected early striatal CCL2 induction in MPTP mice (all colocalized with astrocytes) (Additional file 8: Figure S7), this was much less prominent than in the SNpc. Consequently, for CCR2, while we detected a trend to increased striatal presence of CCR2-GFP^+^ monocytes in MPTP mice, this was much less prominent than in the SNpc and did not reach significance (Additional file 9: Figure S8A), suggesting that CCR2^+^ monocyte infiltration requires the presence of affected neuronal cell bodies or stronger nigro-striatal CCL2 induction.

Thus, by using direct CCR2-GFP labeling, avoiding artifact-prone irradiation/BMT strategies, our results provide strong evidence to answer a long-standing open question—that there is early brain infiltration of inflammatory CCR2^+^ monocytes during DA neurodegeneration, predominantly in the affected SNpc, but in the acute MPTP model, the level of infiltration remained transient and rather limited.

### CCR2 deletion suggests that nigral infiltration of CCR2^+^ monocytes, at the level observed in acute MPTP model mice, does not contribute to loss of DA neurons

**Fig. 5:**
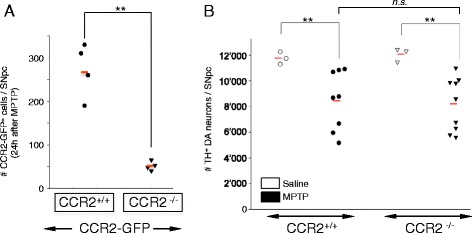
CCR2 deletion blocks nigral CCR2^+^ monocyte infiltration in MPTP mice but does not affect loss of DA neurons. **a** Quantification of the effect of CCR2 deletion on the number of CCR2-GFP^+^ cells infiltrating the SNpc of MPTP treated CCR2-GFP mice. Compared to CCR2-GFP mice with normal CCR2 content (CCR2^+/+^/CCR2-GFP), CCR2 deletion in CCR2-GFP mice (CCR2^−/−^/CCR2-GFP) leads to blockage of nigral infiltration of CCR2-GFP^+^ cells (measured at 24 h after MPTP intoxication). Counts represent the estimated total of CCR2-GFP^+^ cells within the entire SNpc (individual mice are shown; *red bars*, mean; *n* = 4 mice per condition; **P = 0.004, Holm-Sidak method). **b** Quantification of the effect of CCR2-deletion on the amount of DA neuronal loss in MPTP mice. Robust MPTP-induced death of DA neurons is measurable in both wild-type (CCR2^+/+^, *n* = 8) and CCR2 deleted (CCR2^−/−^, *n* = 9) mice (28 and 32%, respectively, compared to saline controls; **P = 0.002, ANOVA with Holm-Sidak test). However, CCR2 deletion does not affect MPTP-induced death of DA neurons (n.s., non-significant, *P* > 0.05). Counts represent the estimated total of TH^+^ DA neurons within the entire SNpc, 7 days after acute MPTP intoxication (individual mice are shown; *red bar*, mean)

To assess whether the limited nigral infiltration of CCR2^+^ monocytes in the acute MPTP model contributes to DA neuronal loss, we used CCR2 deleted mice (CCR2 is required for peripheral tissue infiltration of inflammatory CCR2^+^ blood monocytes) [14, 19]. First, we tested the necessity of a functional CCL2-CCR2 axis for the observed nigral infiltration of CCR2^+^ monocytes in the MPTP model (Fig. 5a). CCR2^−/−^ mice were crossed with CCR2-GFP reporter mice to yield CCR2^−/−^/CCR2-GFP mice. Indeed, compared to control CCR2^+/+^/CCR2-GFP littermates, we found blockage of MPTP-mediated nigral infiltration of CCR2-GFP^+^ cells in CCR2^−/−^ mice (Fig. 5a). Of note, although blockage was almost complete, a few cells remained, representing background or potentially some CCR2-independent perivascular cells.

However, when we determined the effect of blocking this nigral infiltration of CCR2^+^ monocytes on DA neuronal loss at 7 days after acute MPTP intoxication, we did not find any difference between CCR2 deleted (*n* = 9) and wild-type (CCR2^+/+^) (*n* = 8) mice (28% loss of DA neurons and 32%, respectively) (Fig. 5b). Actual counts of DA neurons (as means +/− SEM) were 11,760 +/− 280 (saline, *n* = 3, CCR2^+/+^), 8467 +/− 680 (MPTP, *n* = 8, CCR2^+/+^), 12,080 +/− 320 (saline, *n* = 3, CCR2^−/−^) and 8214 +/− 600 (MPTP, *n* = 9, CCR2^−/−^).

To our knowledge, this is the first time the effect of CCR2 deletion on loss of nigral DA neurons in MPTP mice has been assessed. This provides a clear answer to our initial question—that brain infiltration of CCR2^+^ monocytes does not contribute to loss of DA neurons, at least not in the acute MPTP model of DA neurodegeneration.

The question remains whether the level of CCL2 induction and CCR2^+^ monocyte infiltration in the MPTP model is representative of human PD, where DA neurodegeneration is much more progressive. Our results indicate that either brain infiltrating CCR2^+^ monocytes do not play per se a role during DA neurodegeneration or that in the acute MPTP model, their infiltration remains too limited, not sufficiently high to reveal their actual potential to affect positively or negatively the ongoing DA neuronal loss. Even more tempting, but of more fundamental interest, our findings of early but only transient CCL2 induction and limited CCR2^+^ monocyte infiltration, could suggest an endogenous mechanism that protects DA neurons against too strong induction of CCL2 and deleterious effects of the CCL2-CCR2 axis. In the following, we have assessed these two possibilities.

### Transgenic over-induction of astroytic CCL2 in MPTP mice leads to increased nigral infiltration of CCR2^+^ monocytes and increased loss of dopaminergic neurons, suggesting a neurotoxic potential of such infiltrating CCR2^+^ monocytes

The current lack of efficient tools to test whether and to which level monocytes infiltrate in actual human PD, combined with the limits of the MPTP model to simulate the much more chronic human condition, justify, in our eyes, to further investigate the role of the CCL2-CCR2 axis during DA neurodegeneration.

Therefore, we used the MPTP model and asked *what* the disease contributing effect of such CCR2^+^ monocytes would be, *if* they would infiltrate the SNpc in higher numbers. To achieve this, we used well-characterized transgenic mice that overexpress CCL2 in astrocytes under the astrogliosis sensitive GFAP promotor [33]. In mice, baseline GFAP expression in the brain is heterogeneous, with only weak expression in the SNpc, but strong upregulation during MPTP induced neuroinflammation [3] (Additional file 1: Figure S1). Thus, the use of GFAP-CCL2 mice would lead to increased CCL2 induction during MPTP-mediated DA neurodegeneration, which should increase nigral infiltration of CCR2^+^ monocytes.

**Fig. 6:**
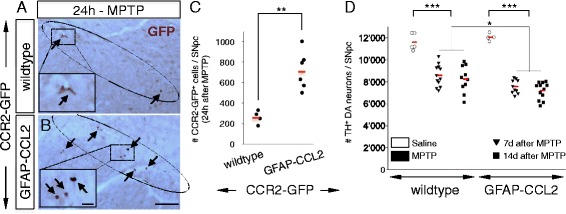
Transgenic over-induction of astrocytic CCL2 in MPTP mice increases nigral CCR2^+^ monocyte infiltration and loss of DA neurons. **a**–**b** Use of transgenic GFAP-CCL2 mice to enhance MPTP-mediated astrocytic CCL2 induction. Immunohistochemistry with anti-*GFP* antibodies (*brown*) shows increased presence of CCR2-GFP^+^ cells (*arrows*) within the MPTP-affected SNpc of GFAP-CCL2/CCR2-GFP double-transgenic mice (**b**) as compared to CCR2-GFP single-transgenic littermates (**a**) (measured at 24 h after MPTP intoxication; *insets* show enlargements). (*Scale bars*; **b**, 200 μm; *inset* in **b**, 10 μm). **c** Quantification of presence of CCR2-GFP^+^ cells within the SNpc (24 h after MPTP intoxication) between GFAP-CCL2/CCR2-GFP mice (*n* = 6) and CCR2-GFP littermates (*n* = 4), suggests that enhancing MPTP-mediated astrocytic CCL2 induction increases nigral CCR2^+^ monocyte infiltration (by 2.7 fold, **P < 0.01, Dunn’s test). Counts represent the estimated total of CCR2-GFP^+^ cells within the entire SNpc (individual mice are shown; *red bars*, mean). **d** MPTP-induced DA neuronal loss in GFAP-CCL2 mice and wild-type non-transgenic littermates. Quantification at 7 (*n* = 12) and 14 days (*n* = 10) after MPTP intoxication, shows robust and stable loss of DA neurons in wild-type mice (26 and 29%, respectively, compared to saline injected controls (*n* = 5); ***P < 0.001, ANOVA with Holm-Sidak test). While saline injected control GFAP-CCL2 mice (*n* = 4) have normal numbers of DA neurons, the MPTP-induced DA neuronal loss compared to wild-type mice is significantly increased in GFAP-CCL2 transgenic mice (*P = 0.01, ANOVA with Holm-Sidak test), both at 7 (*n* = 10) and 14 days (*n* = 12) after MPTP intoxication (37 and 40%, respectively, compared to saline injected controls (*n* = 4); ***P < 0.001, ANOVA with Holm-Sidak test). Counts represent the estimated total of TH^+^ DA neurons within the entire SNpc, 7 and 14 days after acute MPTP intoxication (individual mice are shown; *red bars*, mean)

First, we confirmed that GFAP-CCL2 mice overexpressed CCL2 in the region containing the SNpc (Additional file 10: Figure S9A, D, G). Next, we crossed GFAP-CCL2 mice to CCR2-GFP reporter and indeed found a significantly increased infiltration of CCR2-GFP^+^ monocytes specifically in the affected SNpc during MPTP induced DA neurodegeneration (at 24 h after MPTP intoxication) when comparing double-transgenic GFAP-CCL2/CCR2-GFP mice to single-transgenic CCR2-GFP littermates (Fig. 6a–c and Additional file 10: Figure S9D-I). Of note, saline-injected GFAP-CCL2/CCR2-GFP mice only showed rare CCR2-GFP^+^ cells in the SNpc (Additional file 10: Figure S9A-C).

To assess the effect on neurodegeneration, we quantified DA neuronal loss both at 7 days after MPTP-intoxication but also at 14 days, with the idea that the usually stable neuronal loss might increase due to CCL2 overexpression (Fig. 6d). Importantly, we found a significant increase in DA neuronal loss when comparing mice overexpressing astrocytic CCL2 (GFAP-CCL2) to non-transgenic littermates at both 7 days (37% loss compared to 26%, respectively) and 14 days (40% loss compared to 29%, respectively) (Fig. 6d). While we did not observe a further increase in neuronal loss at 14 days, the difference stayed stable (Fig. 6d). Actual counts of DA neurons (as means +/− SEM) were 11,620 +/− 344 (wild-type, saline, *n* = 5), 8588 +/− 286 (wild-type, MPTP, *n* = 12, 7 days), 8244 +/− 384 (wild-type, MPTP, *n* = 10, 14 days), 12,065 +/− 168 (GFAP-CCL2, saline, *n* = 4), 7574 +/− 194 (GFAP-CCL2, MPTP, *n* = 10, 7 days) and 7203 +/− 288 (GFAP-CCL2, MPTP, *n* = 12, 14 days).

Thus, increasing the number of CCR2^+^ monocytes infiltrating the affected SNpc, suggested that such monocytes, in principal, have a neurotoxic potential in the MPTP model and *could* contribute to DA neurodegeneration, but only if they infiltrate in higher numbers than what is normally present in MPTP mice. We next asked if there is a control mechanism normally limiting the underlying astrocytic CCL2 induction.

### CX3CR1 deletion in MPTP mice leads to CCL2 over-induction in the substantia nigra, suggesting control of astrocytic CCL2 induction by microglial CX3CR1

The above results indicated that a too strong astrocytic CCL2 induction could increase DA neurodegeneration in MPTP mice. A tempting question would be to ask, whether in MPTP mice there is a mechanism that limits such astrocytic CCL2 over-induction, to protect DA neurons against deleterious effects of the CCL2-CCR2 axis.

Interestingly, our previous results from an unrelated neurodegenerative condition, age-related macular degeneration, suggested that deletion of the chemokine receptor CX3CR1 led to deleterious actions of the CCL2-CCR2 axis [34]. In turn, CX3CR1 deleted mice are known to show increased MPTP-mediated loss of nigral DA neurons [30], but the mechanism remains unclear. We hypothesized that this increased neurotoxicity could be *indirectly* driven by astrocytic CCL2 over-induction, likely as a consequence of deregulated reactive responses from CX3CR1 deleted microglia (the main cell type expressing CX3CR1 in the CNS).

**Fig. 7:**
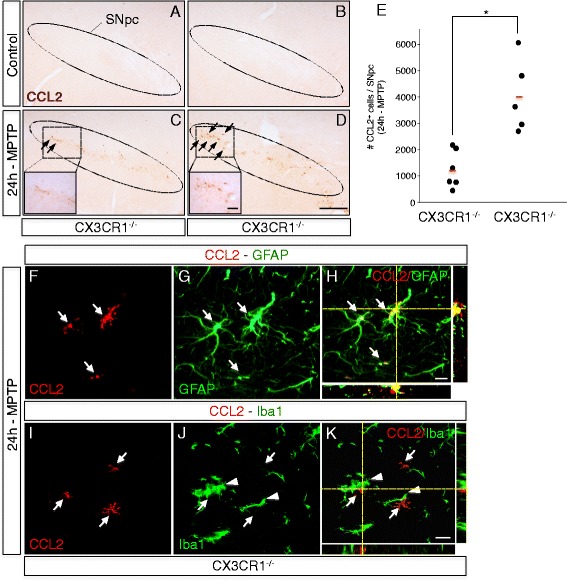
Deletion of microglial CX3CR1 in MPTP mice leads to over-induction of astrocytic *CCL2* in the affected substantia nigra. **a**–**d** Immunohistochemistry with anti-CCL2 antibodies (*arrows*, *brown*) shows enhanced induction of *CCL2* in the MPTP-affected *SNpc* of CX3CR1 deleted mice (*CX3CR1* ^*−/−*^) (**d**) as compared to wild-type littermate controls (*CX3CR1* ^*+/+*^) (**c**) (24 h after intoxication; see *insets* for enlargements). No *CCL2* induction was seen in saline control animals (**a**, **b**). **e** Quantification of increased numbers of CCL2 expressing cells within the MPTP-affected SNpc in *CX3CR1* ^*−/−*^ mice (*n* = 5) as compared to *CX3CR1* ^*+/+*^ littermate controls (*n* = 6), measured at 24 h after intoxication. Counts represent the estimated total cells within the entire *SNpc* (individual mice are shown; *red bars*, mean; quantification, *P = 0.019, Holm-Sidak method). **f**–**k** Immunofluorescence stains of MPTP-affected *SNpc* 24 h after intoxication, showing that in CX3CR1 deleted mice, *CCL2* induction (**f**, **i**; *red*, *arrows*) still colocalized with astrocytes (**g**; *GFAP*, *green*, *arrows*; with confocal view in **h**) and remained absent from microglia (**j**; *Iba1*, *green*, *arrowheads*; with confocal view in **k**). (*Scale bars*; **d**, 100 μm; *insets*, 20 μm; **h** and **k**, 20 μm)

To test this hypothesis, we assessed CCL2 protein induction in the affected SNpc 24 h after MPTP-intoxication in both CX3CR1^−/−^ (*n* = 5) and CX3CR1^+/+^ mice (*n* = 6) (Fig. 7a–e). Of note, CCL2 was absent in (saline) controls from both genotypes (Fig. 7a, b). Consistent with our hypothesis, we found three times more cells inducing CCL2 in the SNpc from CX3CR1^−/−^ than from CX3CR1^+/+^ mice (Fig. 7c–e). This increased CCL2 induction remained astrocytic and was not present in microglia (Fig. 7f–k).

### Increased MPTP-mediated dopaminergic neurodegeneration in CX3CR1-deficient mice is driven by CCL2 over-induction, suggesting that microglial CX3CR1 protects against neurotoxic actions of the CCL2-CCR2 axis

**Fig. 8:**
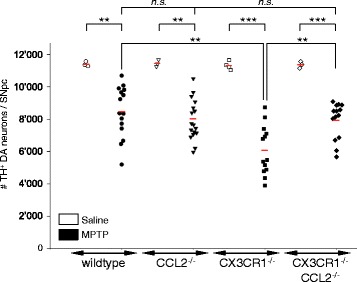
During DA neurodegeneration microglial CX3CR1 protects against neurotoxic CCL2 over-induction by astrocytes. Quantification of the effect of CCL2 deletion on the known increased MPTP-mediated neuronal loss in CX3CR1 deleted mice. Loss of DA neurons was assessed by counting of TH positive DA neurons within the affected SNpc, 7 days after MPTP intoxication. Counts represent the estimated total cells in the entire SNpc (individual mice are shown; *red bars*, mean; quantification, ANOVA with Holm-Sidak test). Compared to wild-type mice (*n* = 15; 26% loss of DA neurons, compared to saline- (*n* = 3) injected controls; **P < 0.01), CX3CR1 deleted mice (CX3CR1^−/−^) show increased loss (**P < 0.01) of DA neurons (*n* = 13; 46% loss of DA neurons, compared to saline (*n* = 4) injected controls; ***P < 0.001), while the loss of DA neurons in CCL2 deleted mice (CCL2^−/−^) (*n* = 17; 30% loss of DA neurons, compared to saline- (*n* = 3) injected controls; **P < 0.01), is not different (*P* > 0.05) from wild-type mice. However, mice with CX3CR1^−/−^/CCL2^−/−^ double deletions (*n* = 14; 30% loss of DA neurons, compared to saline (*n* = 3) injected controls; ***P < 0.001) show less MPTP-induced loss of DA neurons than mice deleted for only CX3CR1 (30% compared to 46% loss of DA neurons; **P < 0.01), resetting the increased level of DA neuronal loss observed in CX3CR1^−/−^ mice (46% loss) to levels observed in normal wild-type mice (26% loss)

To assess whether the increased number of astrocytes inducing CCL2 is directly causative for the more pronounced DA neuronal loss in MPTP intoxicated CX3CR1 deleted mice, we generated mice deficient for both CCL2 and CX3CR1. We then determined the effect on DA neuronal loss 7 days after acute MPTP intoxication. As reported before [30], we confirmed increased DA neuronal loss in single CX3CR1 deleted mice (CX3CR1^−/−^) when compared to wild-type mice (46% loss compared to 26%) (Fig. 8). Of note, single CCL2 deleted mice (CCL2^−/−^) did not show any difference in DA neuronal loss when compared to wild-type mice (30% loss compared to 26%), expected from our data with CCR2 deleted mice (Fig. 5b). Importantly, mice deleted for both CCL2 and CX3CR1 (CX3CR1^−/−^/CCL2^−/−^) showed *less* MPTP-induced loss of DA neurons than mice deleted for only CX3CR1 (CX3CR1^−/−^) (30% loss compared to 46%), resetting the increased level of DA neuronal loss observed in CX3CR1 deleted mice to levels observed in MPTP diseased wild-type mice (Fig. 8). Actual counts of DA neurons (as means +/− SEM) were 11,403 +/− 86 (wild-type, saline, *n* = 3), 8470 +/− 404 (wild-type, MPTP, *n* = 15); 11,451 +/− 125 (CCL2^−/−^, saline, *n* = 3), 8032 +/− 300 (CCL2^−/−^, MPTP, *n* = 17); 11,313 +/− 131 (CX3CR1^−/−^, saline, *n* = 4), 6077 +/− 422 (CX3CR1^−/−^, MPTP, *n* = 13); 11,365 +/− 119 (CX3CR1^−/−^/CCL2^−/−^ saline, *n* = 3) and 7931 +/− 302 (CX3CR1^−/−^/CCL2^−/−^, MPTP, *n* = 14).

## Discussion

We had two aims: On the one hand, to resolve the long-standing questions whether CCR2^+^ monocytes infiltrate the brain in the MPTP model of DA neurodegeneration and whether they contribute to loss of DA neurons. On the other hand, and as a consequence of what we found for monocyte infiltration, to better understand the regulation of the underlying induction of the monocyte-attracting CCL2.

Our initial screen found early nigral induction of the CCL2/7/12-CCR2 axis, suggesting monocyte infiltration. As the microdissection was done on fresh frozen tissues, the CCR2 mRNA increase might have resulted from remaining blood monocytes. However, to avoid this, we have perfused the mice with PBS before tissue collection. Independent of it, the net effect of increased CCR2 in the SNpc of MPTP mice remains specific. On a protein level, CCL2/7 were early induced by nigral astrocytes. This is noteworthy, as classic markers suggest astrogliosis to happen *after* microgliosis [3, 32]. Thus, astrocytes seem to react very early and act as the main source for monocyte-attracting chemokines in this model.

While previous studies have attempted to assess monocyte brain infiltration in MPTP mice, they are unconclusive as they used artifact-prone irradiation/BMT strategies [10–13]. To resolve this, we used CCR2-GFP reporter mice and found early, but transient, nigral infiltration of CCR2^+^ monocytes in acute MPTP mice. However, infiltration was limited and much lower than what was expected from irradiation-based studies. This represents an important finding, as it shows for the first time, with a direct labeling method, whether or not CCR2^+^ monocytes infiltrate the affected SNpc. Although infiltrating CCR2^+^ monocytes will ultimately downregulate CCR2 [19], due to our exhaustive timecourse analysis, we do not think to have underestimated their numbers.

A very recent study also used alternative methods to assess whether myeloid cells infiltrate in MPTP mice [35]. While we used the classic acute intoxication paradigm (four injections during 1 day, [24]), they used the subchronic MPTP model (one daily injection for 5 days, [24]). To label infiltrating cells, they used an indirect labeling strategy (inducible CX3CR1 reporter mice), which allowed to mark microglia without peripheral myeloid cells (due to their higher turnover). By focusing on a single timepoint, they reported nigral infiltration of peripheral myeloid cells, although the exact monocyte cell type was not specified. Comparing the two studies—they report rather significant, we rather limited infiltration—is difficult, due to the different MPTP regiments used, but the results can be interpreted as complementary, considering the known differences between the two regiments. However, the novelity of our study lays in its direct labeling strategy, which allowed to assess infiltration of the most common monocyte subtype, inflammatory-type CCR2^+^ cells, as well as in the precise timecourse analysis for both the nigra and the striatum.

To assess the consequence of the limited nigral infiltration of CCR2^+^ monocytes, we used CCR2 deleted mice. However, while we blocked CCR2^+^ cell infiltration, this did not influence DA neuronal loss in MPTP mice. Our initial RNA screen revealed early peak induction of the monocyte chemokine axis, while the lymphocyte chemokine axis showed more persistent induction. This is consistent with the transient and limited infiltration of CCR2^+^ monocytes (which did not affect DA neuronal loss), as compared to our previous results of more persistent and prominent T lymphocyte infiltration (which was neurotoxic, see [3]). Of note, while we found only limited CCR2^+^ monocyte infiltration, its peak (at 36 h) was slightly before the reported nigral lymphocyte infiltration (at 48 h, see [3]), suggesting that monocyte infiltration happens first. As indicated in the introduction, CCR2-deletion was previously studied in MPTP mice, but without link to monocytes, using a MPTP regiment too low to induce neuronal loss and only striatal DA denervation was analyzed [23]. Of note, while no effect on striatal denervation was reported, this method is much less sensitive than counting actual loss of nigral DA neurons. The MPTP model is very aggressive on striatal denervation (up to 80%), while loss of nigral DA neurons remains moderate (up to 30–40%). Thus, it has been shown that specific genetic modifications had no protective effect on striatal denervation but showed significant protection on nigral neuronal loss [3, 36].

The abovementioned study [35] in the subchronic MPTP model suggested that infiltrating peripheral myeloid cells contribute to DA neurodegeneration via CD95L/CD95 (FasL/Fas). Peripheral myeloid cells were specifically targeted by using irradiation with head protection, followed by bone marrow transplantation from mice with myeloid deletion of CD95L [35]. While a sophisticated strategy, it nevertheless represents a rather invasive approach and focused on a specific pathway.

Importantly, the *global* effect of infiltrating myeloid cells, or more specifically of infiltrating CCR2^+^ monocytes, the focus of *our* question, was not directly addressed. Of note, our previous results also found a deleterious FasL/Fas effect, but in the acute MPTP model, and mediated by infiltrating T lymphocytes [3].

One further study assessed brain infiltrating monocytes in MPTP mice [37], but the main focus was rather on the enteric nervous system. They used clodronate liposomes to transiently deplete blood monocytes and found a protective effect on myenteric and no effect on brain DA neurodegeneration. However, the MPTP regiment used was not sufficient to induce loss of nigral DA neurons, only striatal denervation was assessed (*see comments above*) and monocyte depletion was partial (preferentially phagocytic active ones). While the study found no striatal infiltration, this was only indirectly measured via density of microglial/macrophage markers. Thus, this study could not answer our question regarding the presence and role of brain infiltrating CCR2^+^ monocytes on DA neuronal loss. Of note, the study found a direct MPTP effect on blood monocytes, which was previously also suggested for lymphocytes [38]. However, it can be assumed that the primary cause for MPTP neurotoxic derives from a direct effect on the DA neurons.

Thus, despite previous efforts, the question of whether brain infiltrating CCR2^+^ monocytes do contribute to neuronal death in a model of DA neurodegeneration, remained open. We believe that our straight-forward approach of CCR2-GFP reporter mice and CCR2-deletion provides now a clear answer: The limited nigral infiltration of CCR2^+^ monocytes in acute MPTP mice does not contribute to loss of DA neurons.

While MPTP mice are a powerful tool to analyze DA neurodegeneration, it remains a very rapid acting model compared to progressive human PD. Our results showed limited infiltration and no effect on neuronal loss in mice. Actual brain monocyte infiltration in humans (or mice) might only be present during such longer chronic states. However, the situation in humans remains elusive, as, in contrast to infiltrating lymphocytes, the tools to detect infiltrating monocytes are lacking. Of note, in the human blood, the ratio of inflammatory-type (classical/CCR2^+^) monocytes to patrolling-type (non-classical/CCR2^−^) monocytes is much higher than in mice [20], which might influence the level of a potential brain infiltration during human disease. Together with recent data suggesting increased serum CCL2 levels and deregulated CCR2^+^ blood monocytes responses in PD patients [39, 40], it remains possible that brain infiltrating monocytes are relevant for human disease, despite our negative results from the acute mouse model. Furthermore, although CNS infiltrating CCR2^+^ monocytes are assumed to rather aggravate neurodegeneration/injury, this seems not that clear-cut: While they act deleterious e.g., in traumatic brain injury models [41, 42], they are protective in motor neuron disease ALS mice [43].

Thus, we used transgenic astrocytic CCL2 overexpression to increase CCR2^+^ monocyte infiltration and found that this increased death of DA neurons in MPTP mice. While it remains artificial, this suggests that CCR2^+^ monocytes have, in principal, a neurotoxic potential and *could* contribute to DA neurodegeneration. However, in normal mice, acute MPTP intoxication does not lead to deleterious high levels of CNS monocyte infiltration. This raised the question, whether there is a mechanism that limits the underlying astrocytic CCL2 induction, to avoid deleterious CCL2-CCR2 axis effects. Interestingly, based upon our own previous results from age-related macular degeneration [34, 44], we found evidence suggesting that this involves microglial CX3CR1 signaling. CX3CR1 deleted mice serve as a model, in which light-induction leads to photoreceptor degeneration, while normal mice show less degeneration [44]. Compared to normal mice, retinal degeneration in CX3CR1^−/−^ mice was paralleled by *increased* CCL2 induction and retinal CCR2^+^ monocytes infiltration [34]. CCR2 deletion blocked retinal degeneration, suggesting that CX3CR1 usually represses CCL2 over-induction and recruitment of neurotoxic levels of CCR2^+^ monocytes [34]. Transferring this to DA neurodegeneration, it is well known that CX3CR1 deleted mice show increased MPTP-induced DA neuronal loss [30]. While this has been attributed to deregulated neuron-microglia interactions, we speculated—based on our question to find regulators of CCL2 induction—that it could *indirectly* be due to neurotoxic CCL2 over-induction. While such CCL2 over-induction could be a simple consequence of increased neurodegeneration, when we generated a CX3CR1/CCL2 double deletion, we rescued the initial increased neuronal loss in MPTP mice, indicating that CCL2 over-induction is causative. Thus, we have found unexpected evidence for a potential regulation of CCL2 induction by CX3CR1-signaling. This suggests that during DA neurodegeneration, microglial CX3CR1 protects against astrocytic CCL2 over-induction, which otherwise would become deleterious and would increase death of DA neurons. However, as we base our interpretation solely on the analysis of single CX3CR1-KO, CCL2-KO, and double CCL2/CX3CR1-KO mice, potential compensatory effects [45] could play into the observed result. Thus, our proposed regulation of astrocytic CCL2 induction by microglial CX3CR1, remains of correlative nature.

In the CNS, the most likely CCL2 target are brain infiltrating CCR2^+^ monocytes. When we increased their infiltration in MPTP mice using transgenic CCL2 over-induction, death of DA neurons was increased. Although it remains again purely correlative, this could suggest that microglial CX3CR1 signaling, by limiting astrocytic CCL2 over-induction, could protect against neurotoxic levels of brain infiltrating CCR2^+^ monocytes. This could be the reason for the only limited nigral CCR2^+^ monocyte infiltration we detected in wild-type MPTP mice, which did not affect loss of DA neurons. However, while the principal receptor is CCR2, there might be other low efficiency receptors, which could mediate the neurotoxic effect of CCL2 over-induction. Likewise, while in the brain CCR2 is mainly expressed by infiltrating monocytes, other cell types (even neurons) might express low CCR2 levels (not detected by CCR2-GFP) and mediate the neurotoxic effect of CCL2 over-induction. Of note, CCR2 can also be expressed by a (minor) subpopulation of T lymphocytes (subsets of Treg cells) [46]; however, all CCR2-GFP^+^ cells we detected in the affected SNpc were also positive for CD11b or Iba1, suggesting monocytes.

Interestingly, CX3CR1^−/−^ microglia are known to produce higher IL-1β levels after LPS induction [30]. In turn, IL-1β can induce CCL2 in cultured astrocytes [47], suggesting a possible mechanism for the astrocytic CCL2 over-induction we detected in CX3CR1 deleted MPTP mice. Of note, in our initial RNA screen in the SNpc of wild-type MPTP mice (without CCL2 over-induction), *Il1b* was actually included as a general inflammatory marker and showed a moderate induction (Additional file 2: Table S1C). Of note, in a mouse model of glioblastoma, CX3CR1 deficiency led to increased CNS infiltration of CCR2^+^ monocytes into the tumor tissue, and it was suggested that this could be linked to increased microglial IL-1β, which in turn could induce CCL2 by tumor cells [48].

The main focus of our analysis was the substantia nigra, but we have also collected data from the striatum. Interestingly, we found only rare astrocytic CCL2 induction in the striatum and no evidence for increased striatal presence of infiltrating CCR2^+^ monocytes in MPTP mice. However, this was with normal MPTP mice. Surprisingly, we have the first evidence that in both transgenic lines with increased MPTP-induced DA neuronal loss, we found similar effects in both nigra and striatum: Compared to normal MPTP mice, in CCL2 overexpressing mice, we found increased presence of striatal CCR2^+^ monocytes (Additional file 9: Figure S8B), and in CX3CR1 deleted mice, we found clear induction of astrocytic CCL2 in the striatum (Additional file 9: Figure S8C). While this remains speculative, it could suggest that induction of the CCL2-CCR2 axis only contributes to DA neurodegeneration, when it is present around both the cell bodies and the target region of affected DA neurons.

## Conclusions

We found that during DA neurodegeneration, there is early induction of CCL2, the main chemokine to attract CCR2^+^ monocytes, and that the major source within the affected nigra are astrocytes. By using CCR2-GFP reporter mice and CCR2-deletion, we applied straight-forward methods to provide answers to two long-standing questions: In the acute MPTP model of DA neurodegeneration, there is limited nigral infiltration of CCR2^+^ monocytes and they do not contribute to loss of DA neurons. However, by analyzing the regulation of the underlying CCL2 induction, we found that increasing CCL2 induction became neurotoxic. Importantly, we found surprising evidence suggesting that during DA neurodegeneration, microglial CX3CR1-signaling protects against such neurotoxic over-induction of CCL2 by astrocytes. This could hint at a mechanism to limit neurotoxic actions of the CCL2-CCR2 axis, including potentially neurotoxic high levels of nigrostriatal CCR2^+^ monocyte infiltration.

## Additional files

Additional file 1: Figure S1. Global neuroinflammation and neurodegeneration in the substantia nigra of acute MPTP model mice. (*A-C*) Immunohistochemical stainings (anti-TH; brown) of DA neuronal degeneration at 2d (*B*) and loss at 7d (*C*) after acute MPTP intoxication in wild-type mice (C57BL/6J). Quantification shows the expected stable loss of around 30% of DA neurons (11,680 +/− 240, saline, *n* = 3; 8410 +/− 440, MPTP, *n* = 10) (28% loss; *P* < 0.05, Holm-Sidak method). Immunofluorescence stainings of (*D-F*) microglial activation (Iba1, red) in the SNpc during MPTP-mediated DA neurodegeneration (TH, green), showing clear signs of microglial reactivity already at 2 days after MPTP intoxication (before the peak of DA neuronal loss, at 4 days) and which is further increased until 7 days (after the peak of DA neuronal loss, when neuronal death has reached a stable level). (*G-I*). Astroglial activation, as measured by GFAP activation (GFAP, red), is delayed compared to microglial activation, only getting strongly induced between 2–7 days after MPTP intoxication. (Scale bar: *F*, 200 μm). (PDF 3173 kb)
Additional file 2: Table S1. RNA profiling of chemokines in the laser-microdissected substantia nigra of the MPTP mouse model. Shown are results from the RT-qPCR profiling of 96 genes using TaqMan 384-well microfluidic cards, to assess all 61 chemokine family members (37 ligands, 24 receptors) as well as 32 other selected neuroinflammation-linked genes, normalized to *Hprt1*. RNA profiling was done in laser-microdissected SNpc at 2, 4, and 7 days after acute MPTP intoxication in adult 12 weeks old C57BL/6J males and compared to saline-injected controls (*n* = 9 mice per condition; as biological replicates). qPCR results are shown as normalized and averaged fold-changes (FC) to saline controls. In addition, the raw averaged C_t_ values of both the control and the 2 days (MPTP) samples are shown. C_t_ values of >30 are called absent (*Abs*). Only FC values that are statistically significant (*P* < 0.05; qBasePlus, Biogazelle) are indicated. Of note, the large FC values can be explained by both the enrichment provided by the LMD and the often very low expression in baseline control conditions. (*nc*, expressed but no significant change and *N.D*., non-determined) (** asterisks* mark unclear corresponding receptors; Griffith et al*.* 2014). (PDF 163 kb)
Additional file 3: Figure S2. RNA profiling of the full chemokine family in the laser-microdissected substantia nigra of MPTP mice. (*A*) Representative example of a RNA integrity analysis of RNA isolated from laser-microdissected SNpc regions (50 per mouse, resulting in 50 ng of total RNA) of fresh frozen Nissl-stained sections and showing sufficient quality RNA for qPCR analysis (RIN >7; BioAnalyzer, PicoAssay II). (*B*) Results show all the chemokines (ligands only) found to be regulated (TaqMan RT-qPCR arrays; at 2, 4, and/or 7 days after acute MPTP intoxication) in the SNpc during DA neurodegeneration (27 of the 37 chemokines tested). Four regulation profiles (Additional file 2: Table S1B) can be distinguished: (“*profile A*”)*:* 10 chemokines showing strong early peak induction at 2 days, then less strong induction at 4 and 7 days (*Ccl2/3/4/7/8/12/22*, *Cxcl2/10/11*); (“*profile B*”)*:* 10 chemokines showing early but persistent induction (at 2/4 and 7 days) (*Ccl5/9/11/19*, *Cxcl1/5-6/4/9/14/16*); (“*profile C*”*):* 2 chemokines showing increased upregulation at the late timepoint (*Ccl6*, *xCl1*), and (“*profile D*”): 4 chemokines showing persistent (*Ccl20/28*) or late downregulation (*Ccl17/Cxcl13*). In addition, one chemokine (CCL24) showed a mixed regulation (early down, then upregulated) (Additional file 2: Table S1B). (PDF 505 kb)
Additional file 4: Figure S3. CCL2 deleted mice confirm high specificity of the used anti-CCL2 antibody. (*A-D*) Immunofluorescence stainings of LPS-injected midbrain region (48 h after injection, known to produce a strong neuroinflammatory reaction which induces CCL2) to assess anti-CCL2 antibody specificity. (*A*, *C*) Results show strong CCL2 positive signal (red), partially colocalizing with astrocytes (GFAP, green; inset in *C*) in wild-type mice (CCL2^+/+^), while this CCL2 signal is completely absent in CCL2 deleted mice (CCL2^−/−^) (*B*, *D*), demonstrating specificity of the anti-CCL2 antibody used. (*Scale bars*; *C*, 200 μm; *C*’, 50 μm). (PDF 3822 kb)
Additional file 5: Figure S4. Induction of the additional two CCR2 ligands, astrocytic CCL7 and microglial CCL12, within the affected substantia nigra of MPTP mice. (*A-F*) Immunofluorescence stainings to show that CCL7 (a ligand for CCR2, like CCL2) (red) is not induced under control saline conditions in the SNpc (dotted white line) (*A*, *D*) but gets induced (red, arrows) 24 h after MPTP intoxication (peak induction) in selected cells, that are not microglia (Iba1, green; arrowheads; with enlargement in *C’*) (*B*, *C*), but astrocytes (GFAP, green, arrows) (*E-F*) (with a confocal orthogonal view in *F*’). (*G-L*) Immuno stainings to show that CCL12 (a ligand for CCR2, like CCL2) (red) is not induced under control saline conditions in the SNpc (dotted white line) (*G*) but gets induced (red, arrows) 24 h after MPTP intoxication (peak induction) in microglia (*H-I*) (Iba1, green; with enlargements in *I*’). This is also shown using confocal analysis (*J-L*). (Scale bars; *F*, *I*, 200 μm; *C*’, *I*’, 40 μm; *F*’, *L*, 20 μm). (PDF 4791 kb)
Additional file 6: Figure S5. Immunostaining characteristics of secreted (CCL2/12) and membrane-bound (CXCL16) chemokines identified in the affected substantia nigra of MPTP mice. (*A-I*) Immunofluorescence stainings to provide support for the specificity of the identified *vesicular* staining-patterns detected for the secreted chemokines CCL2 (red, arrow) in astrocytes (*A-C*) (GFAP, green; with confocal analysis in *C*’) and CCL12 (red, arrows) (*D-F*) in microglia (Iba1, green; TH, blue; with confocal analysis in *F*’), by comparing to the *membranous* staining-pattern detected for the membrane-bound chemokine CXCL16 (*G-I*) in microglia (Iba1, green; TH, blue; with confocal analysis in *I*’). (Scale bars; *C*, *F*, *I*, 20 μm). (PDF 1781 kb)
Additional file 7: Figure S6. CCR2^+^ monocytes infiltrating the affected substantia nigra in MPTP mice are present both perivascular and parenchymal. (*A-F*) Immunofluorescence stainings within the SNpc (anti-TH, blue) of CCR2-GFP mice, 24 h after MPTP intoxication to show both (*A-C*) CCR2-GFP^+^ cells (anti-GFP, green, arrows) with a localization at blood vessel endothelial cells (lectin, red, arrowheads), as well as (*D-F*) with a localization within the parenchyma, consistent with recently infiltrated CCR2-GFP^+^ monocytes. Of note, lectin can also mark microglia, but at the concentration used, mostly vessels. (*G-H*) Confirmatory stainings to show a specific vessel marker (anti-Collagen-IV; red, arrowheads), with parenchymal localization of CCR2-GFP^+^ monocytes in the SNpc (GFP; green, arrows). (Scale bars: *F*, *H*, 50 μm). (PDF 3580 kb)
Additional file 8: Figure S7. Induction of CCL2 within astrocytes in the striatum of MPTP mice. (*A*) Schematic drawing of the nigro-striatal pathway of DA neurons. (*B-F*) Immunofluorescence stainings showing astrocytic localization (GFAP; green, arrow) of CCL2 induction (red, arrows) in the striatum (TH, DA innervation; blue) at 24 h after MPTP intoxication (no staining was detectable in saline injected mice, *data not shown*). (*B*) Example of a cytoplasmic CCL2 staining (shown as a confocal analysis in *D-F*). (*C*) Rare example of a potential extracellular CCL2 staining (red, arrowheads; with DAPI as a nuclear marker, blue) in close proximity to astrocytic processes (GFAP; green, arrows); only detected in the striatum, not in the SNpc. Of note, CCL2 induction was less prominent in the striatum (including at later timepoints than 24 h, *data not shown*) than in the SNpc, (*see* Figs. 2 and 3). (Scale bars; *B*, *C*, *F*, 20 μm). (PDF 1646 kb)
Additional file 9: Figure S8. Quantitative analysis of presence of CCR2^+^ monocytes and induction of CCL2 within the striatum of MPTP mice. (*A*) Full timecourse analysis of the presence of CCR2-GFP^+^ cells (inset left; GFP, brown) within the striatum of MPTP treated CCR2-GFP mice (from 12 h to 7 days after MPTP intoxication). CCR2-GFP^+^ cells (inset right) were double-positive for GFP (green) and cd11b (red), identifying them as monocytes. While there was a trend for an increase at 36 h after MPTP intoxication, this did not reach statistical significance (n.s.; *P* > 0.05; Kruskal-Wallis test). Of note, in contrast to the striatum, *in the substantia nigra*, an increased CCR2^+^ monocyte infiltration was measured at 36 h after intoxication (see, Fig. 4d). Counts represent average CCR2-GFP^+^ cells within a striatal section (means +/− SEM; 5–10 section counted; *n* = 4 mice per condition). (Scale bar; *A*, 10 μm). (*B*) Analysis of striatal CCR2^+^ monocyte infiltration in MPTP mice with transgenic over-induction of astrocytic CCL2. A significant increase in striatal CCR2^+^ monocyte infiltration was measured (at 24 h after MPTP intoxication) in CCR2-GFP/GFAP-CCL2 mice as compared to CCR2-GFP littermates (*n* = 4 mice per condition; **P* < 0.05; *t* test). Of note, this was comparable to the significant increased CCR2^+^ monocyte infiltration that was detectable (at 24 h after intoxication) *in the substantia nigra* of CCR2-GFP/GFAP-CCL2 mice (see, Fig. 6c). (*C*) Analysis of striatal astrocytic CCL2 induction in MPTP mice with deletion for CX3CR1. A significant increased numbers of astrocytes inducing CCL2 was measured (at 24 h after intoxication) in CX3CR1^−/−^ mice as compared to wild-type CX3CR1^+/+^ littermates (*n* = 4 mice per condition; **P* < 0.05; *t* test). Of note, this was comparable to the significant increased numbers of astrocytes inducing CCL2 that was detectable *in the substantia nigra* of CX3CR1-deleted mice (see, Fig. 7e). (PDF 682 kb)
Additional file 10: Figure S9. Confirmation of CCL2 overexpression in GFAP-CCL2 mice. (*A-C*) Immunofluorescence stainings showing that baseline overexpression of CCL2 (red) in control (saline-injected) GFAP-CCL2/CCR2-GFP double-transgenic mice is mostly localized around, but less within the SNpc *(A)*, which is marked with TH (blue). Only very rare CCR2-GFP^+^ cells (green) are detected under baseline conditions in or around the SNpc (*B*). (*D-I*) Strong CCL2 induction (red) is detected in the SNpc (marked with TH, blue) of MPTP treated GFAP-CCL2/CCR2-GFP double transgenic mice (*D* and inset, *G*), leading to increased presence of CCR2-GFP^+^ cells (green) in the affected SNpc (*E-F* and insets, *H-I*) (measured at 24 h after intoxication). (Scale bars; *B*, 200 μm; *I*, 20 μm). (PDF 1619 kb)

## Abbreviations

BAC: Bacterial artificial chromosome
BMT: Bone marrow transplantation
CNS: Central nervous system
DA: Dopaminergic
GFP: Green fluorescent protein
LMD: Laser microdissection
LPS: Lipopolysaccharides
MCP-1/*Ccl2*: Monocyte chemoattractant protein 1
MPTP: 1-Methyl-4-phenyl-1,2,3,6-tetrahydropyridine
RIN: RNA integrity number
RT-qPCR: Reverse transcription quantitative PCR
SNpc: Substantia nigra *pars compacta*
TH: Tyrosine hydroxylase
