# Supplementary figures and images for: Analysis of monocyte infiltration in MPTP mice reveals that microglial CX3CR1 protects against neurotoxic over-induction of monocyte-attracting CCL2 by astrocytes
Source: J Neuroinflammation. 2017 Mar 21;14:60. doi: 10.1186/s12974-017-0830-9 (PMC5359822; doi:10.1186/s12974-017-0830-9)

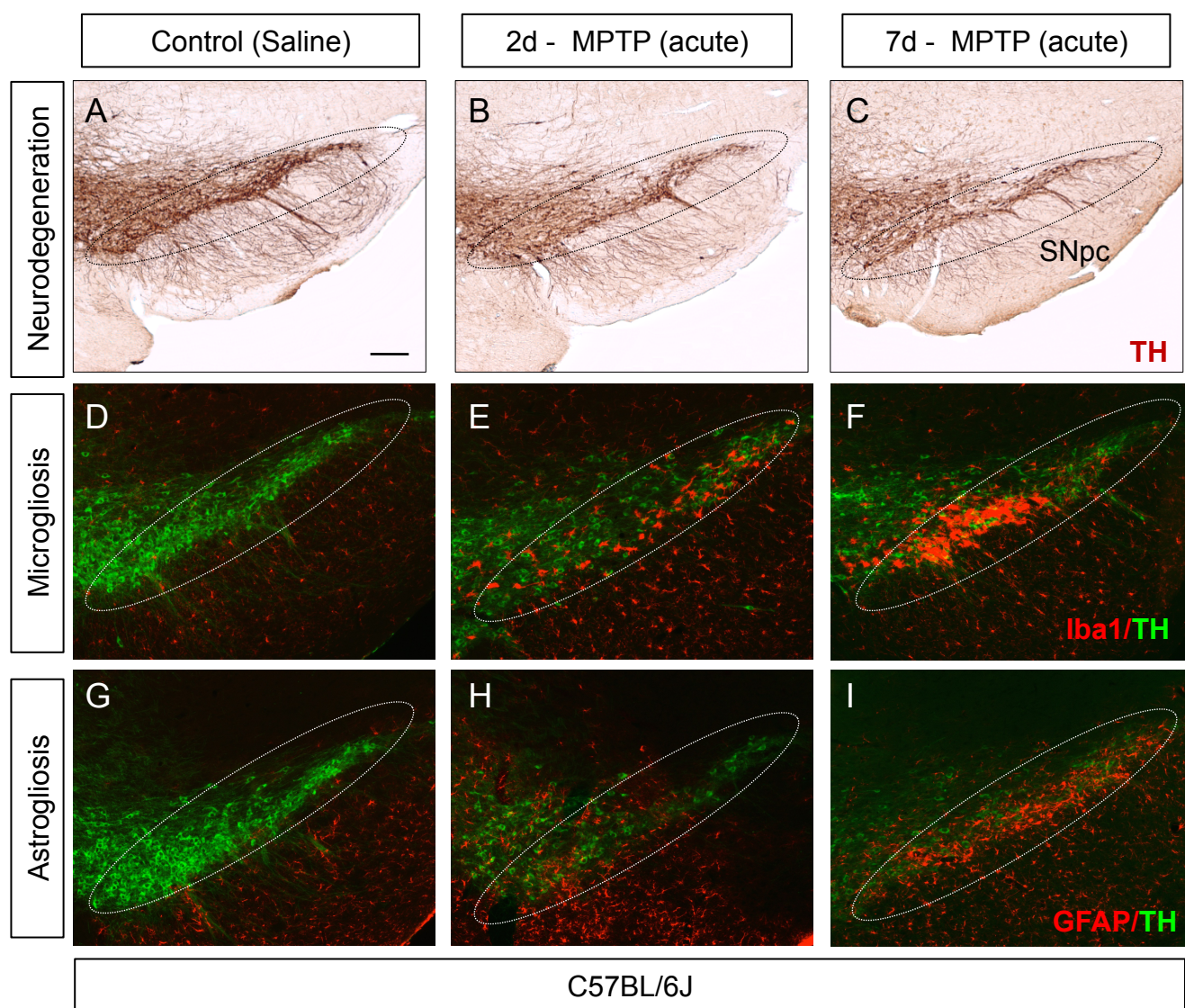

Suppl. Figure 1.

Supplement: Additional file 1: Figure S1. — Global neuroinflammation and neurodegeneration in the substantia nigra of acute MPTP model mice. (A-C) Immunohistochemical stainings (anti-TH; brown) of DA neuronal degeneration at 2d (B) and loss at 7d (C) after acute MPTP intoxication in wild-type mice (C57BL/6J). Quantification shows the expected stable loss of around 30% of DA neurons (11,680 +/− 240, saline, n = 3; 8410 +/− 440, MPTP, n = 10) (28% loss; P < 0.05, Holm-Sidak method). Immunofluorescence stainings of (D-F) microglial activation (Iba1, red) in the SNpc during MPTP-mediated DA neurodegeneration (TH, green), showing clear signs of microglial reactivity already at 2 days after MPTP intoxication (before the peak of DA neuronal loss, at 4 days) and which is further increased until 7 days (after the peak of DA neuronal loss, when neuronal death has reached a stable level). (G-I). Astroglial activation, as measured by GFAP activation (GFAP, red), is delayed compared to microglial activation, only getting strongly induced between 2–7 days after MPTP intoxication. (Scale bar: F, 200 μm). (PDF 3173 kb) [file 12974_2017_830_MOESM1_ESM.pdf]

**A**

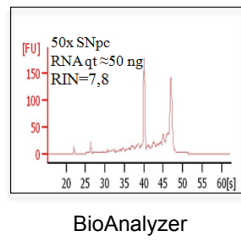

**B**

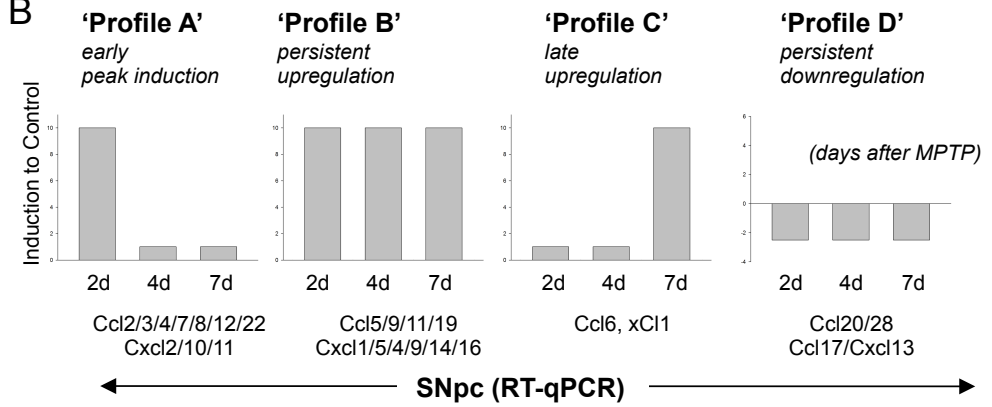

**Suppl. Figure 2.**

Supplement: Additional file 3: Figure S2. — RNA profiling of the full chemokine family in the laser-microdissected substantia nigra of MPTP mice. (A) Representative example of a RNA integrity analysis of RNA isolated from laser-microdissected SNpc regions (50 per mouse, resulting in 50 ng of total RNA) of fresh frozen Nissl-stained sections and showing sufficient quality RNA for qPCR analysis (RIN >7; BioAnalyzer, PicoAssay II). (B) Results show all the chemokines (ligands only) found to be regulated (TaqMan RT-qPCR arrays; at 2, 4, and/or 7 days after acute MPTP intoxication) in the SNpc during DA neurodegeneration (27 of the 37 chemokines tested). Four regulation profiles (Additional file 2: Table S1B) can be distinguished: (“profile A”): 10 chemokines showing strong early peak induction at 2 days, then less strong induction at 4 and 7 days (Ccl2/3/4/7/8/12/22, Cxcl2/10/11); (“profile B”): 10 chemokines showing early but persistent induction (at 2/4 and 7 days) (Ccl5/9/11/19, Cxcl1/5-6/4/9/14/16); (“profile C”): 2 chemokines showing increased upregulation at the late timepoint (Ccl6, xCl1), and (“profile D”): 4 chemokines showing persistent (Ccl20/28) or late downregulation (Ccl17/Cxcl13). In addition, one chemokine (CCL24) showed a mixed regulation (early down, then upregulated) (Additional file 2: Table S1B). (PDF 505 kb) [file 12974_2017_830_MOESM3_ESM.pdf]

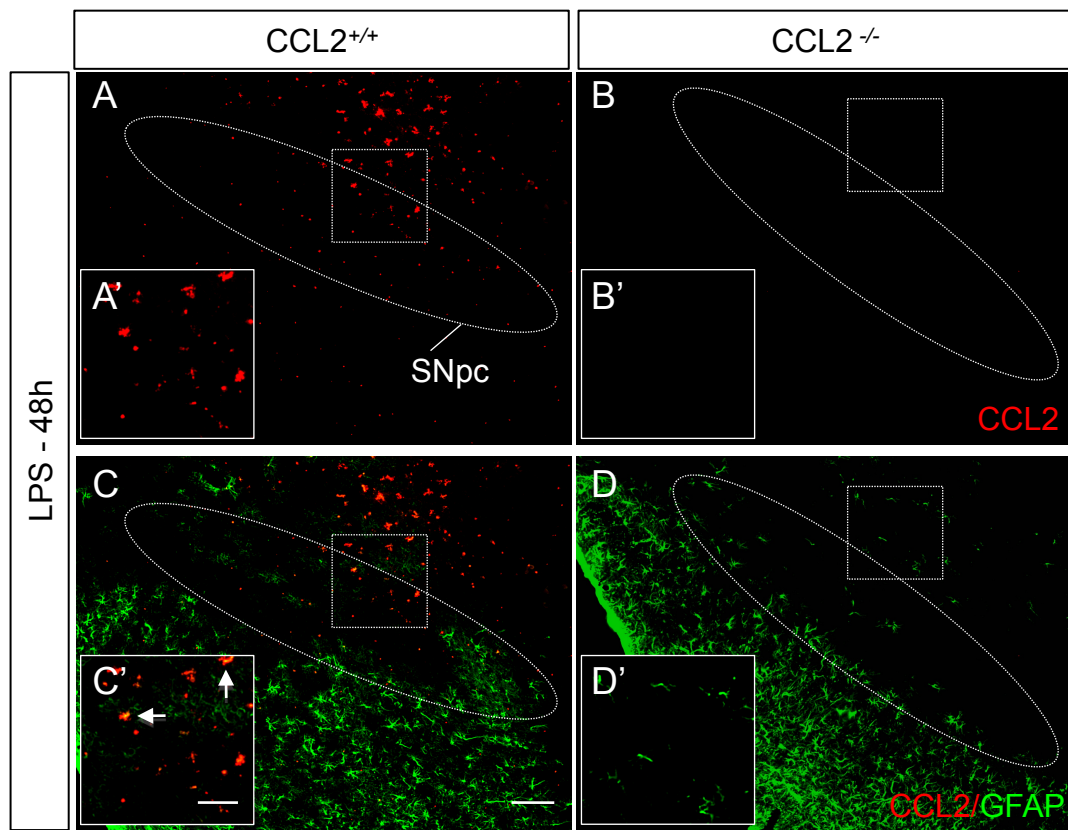

Suppl. Figure 3.

Supplement: Additional file 4: Figure S3. — CCL2 deleted mice confirm high specificity of the used anti-CCL2 antibody. (A-D) Immunofluorescence stainings of LPS-injected midbrain region (48 h after injection, known to produce a strong neuroinflammatory reaction which induces CCL2) to assess anti-CCL2 antibody specificity. (A, C) Results show strong CCL2 positive signal (red), partially colocalizing with astrocytes (GFAP, green; inset in C) in wild-type mice (CCL2+/+), while this CCL2 signal is completely absent in CCL2 deleted mice (CCL2−/−) (B, D), demonstrating specificity of the anti-CCL2 antibody used. (Scale bars; C, 200 μm; C’, 50 μm). (PDF 3822 kb) [file 12974_2017_830_MOESM4_ESM.pdf]

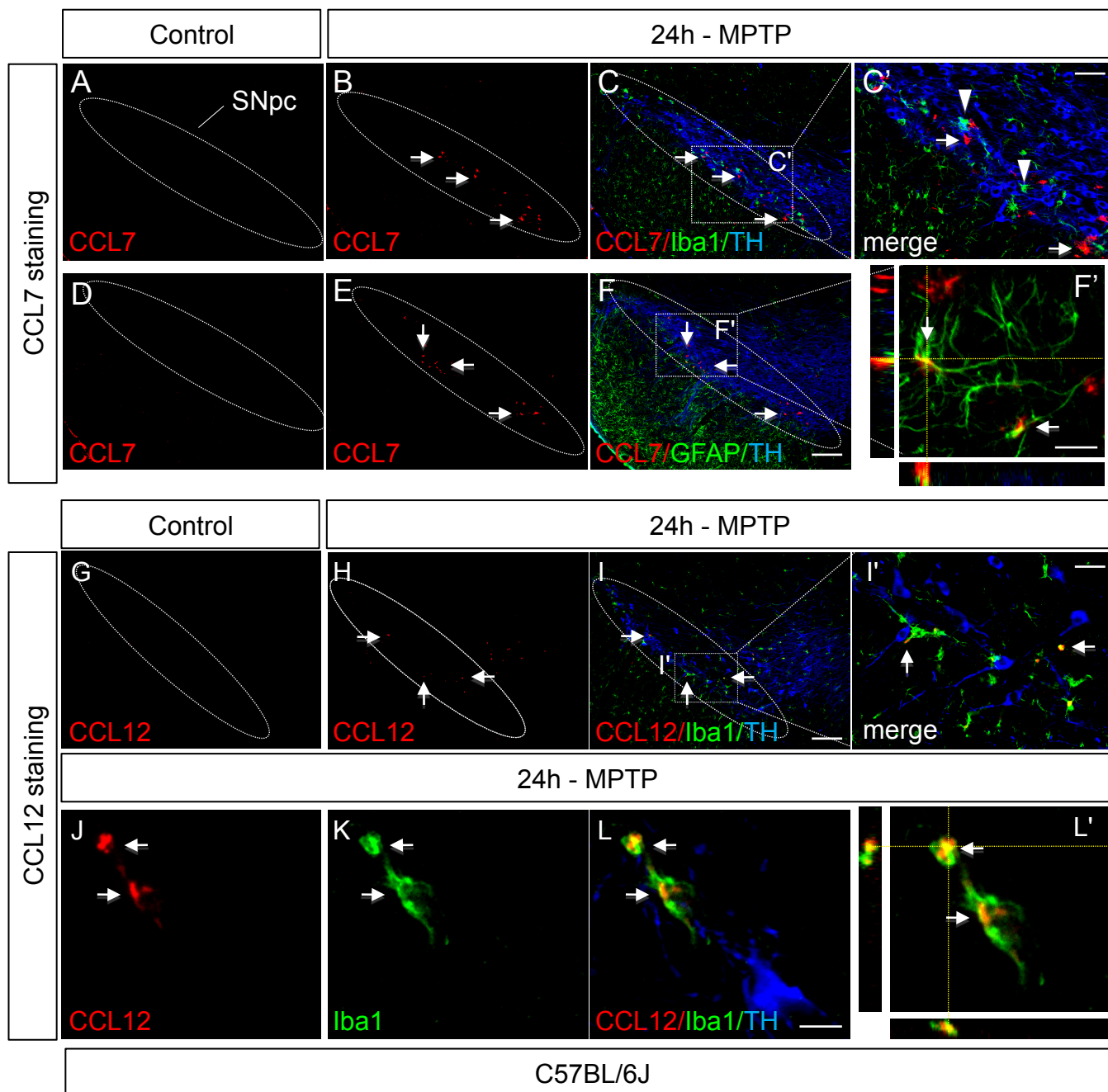

Suppl. Figure 4.

Supplement: Additional file 5: Figure S4. — Induction of the additional two CCR2 ligands, astrocytic CCL7 and microglial CCL12, within the affected substantia nigra of MPTP mice. (A-F) Immunofluorescence stainings to show that CCL7 (a ligand for CCR2, like CCL2) (red) is not induced under control saline conditions in the SNpc (dotted white line) (A, D) but gets induced (red, arrows) 24 h after MPTP intoxication (peak induction) in selected cells, that are not microglia (Iba1, green; arrowheads; with enlargement in C’) (B, C), but astrocytes (GFAP, green, arrows) (E-F) (with a confocal orthogonal view in F’). (G-L) Immuno stainings to show that CCL12 (a ligand for CCR2, like CCL2) (red) is not induced under control saline conditions in the SNpc (dotted white line) (G) but gets induced (red, arrows) 24 h after MPTP intoxication (peak induction) in microglia (H-I) (Iba1, green; with enlargements in I’). This is also shown using confocal analysis (J-L). (Scale bars; F, I, 200 μm; C’, I’, 40 μm; F’, L, 20 μm). (PDF 4791 kb) [file 12974_2017_830_MOESM5_ESM.pdf]

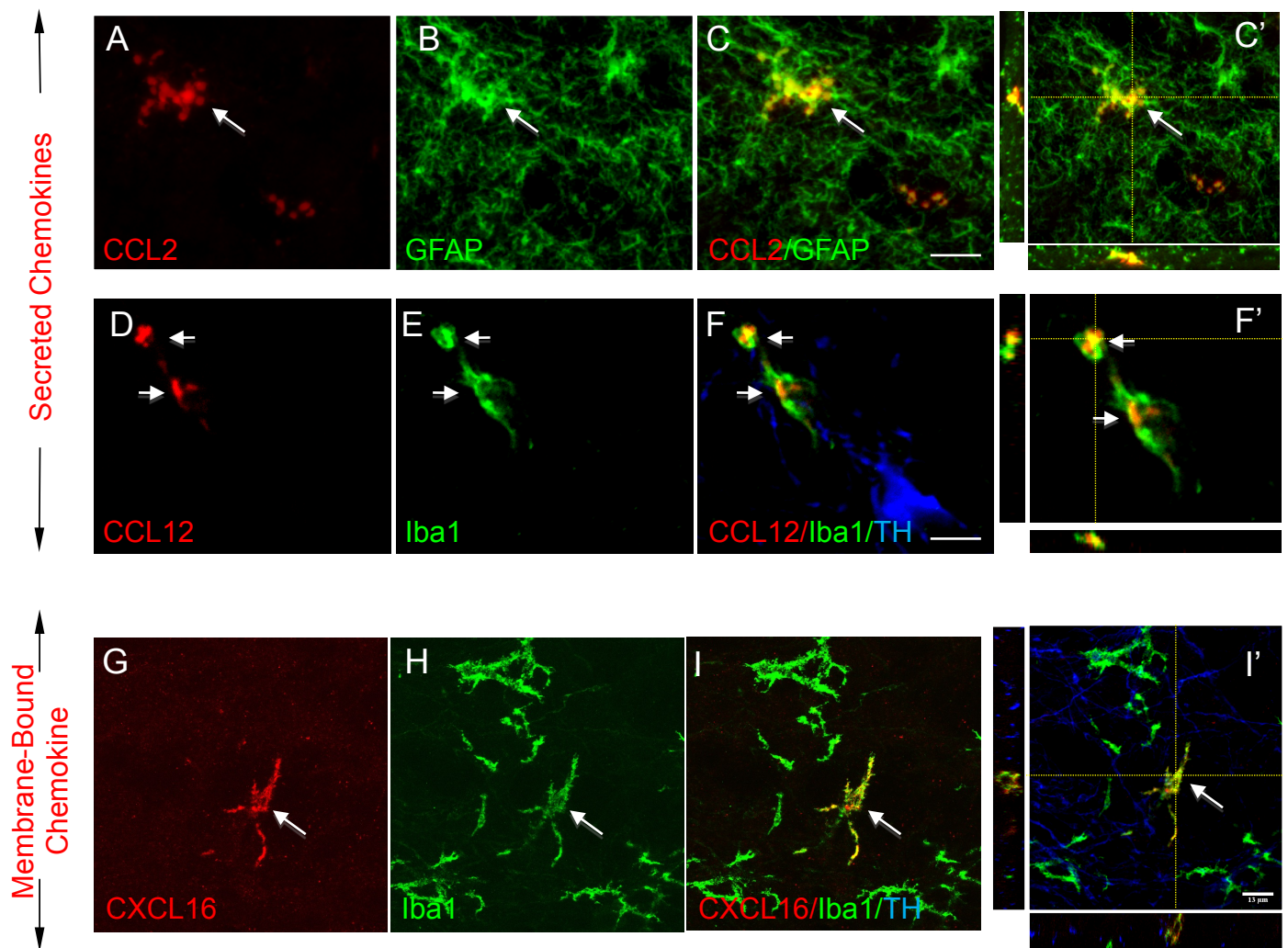

Suppl. Figure 5:

Supplement: Additional file 6: Figure S5. — Immunostaining characteristics of secreted (CCL2/12) and membrane-bound (CXCL16) chemokines identified in the affected substantia nigra of MPTP mice. (A-I) Immunofluorescence stainings to provide support for the specificity of the identified vesicular staining-patterns detected for the secreted chemokines CCL2 (red, arrow) in astrocytes (A-C) (GFAP, green; with confocal analysis in C’) and CCL12 (red, arrows) (D-F) in microglia (Iba1, green; TH, blue; with confocal analysis in F’), by comparing to the membranous staining-pattern detected for the membrane-bound chemokine CXCL16 (G-I) in microglia (Iba1, green; TH, blue; with confocal analysis in I’). (Scale bars; C, F, I, 20 μm). (PDF 1781 kb) [file 12974_2017_830_MOESM6_ESM.pdf]

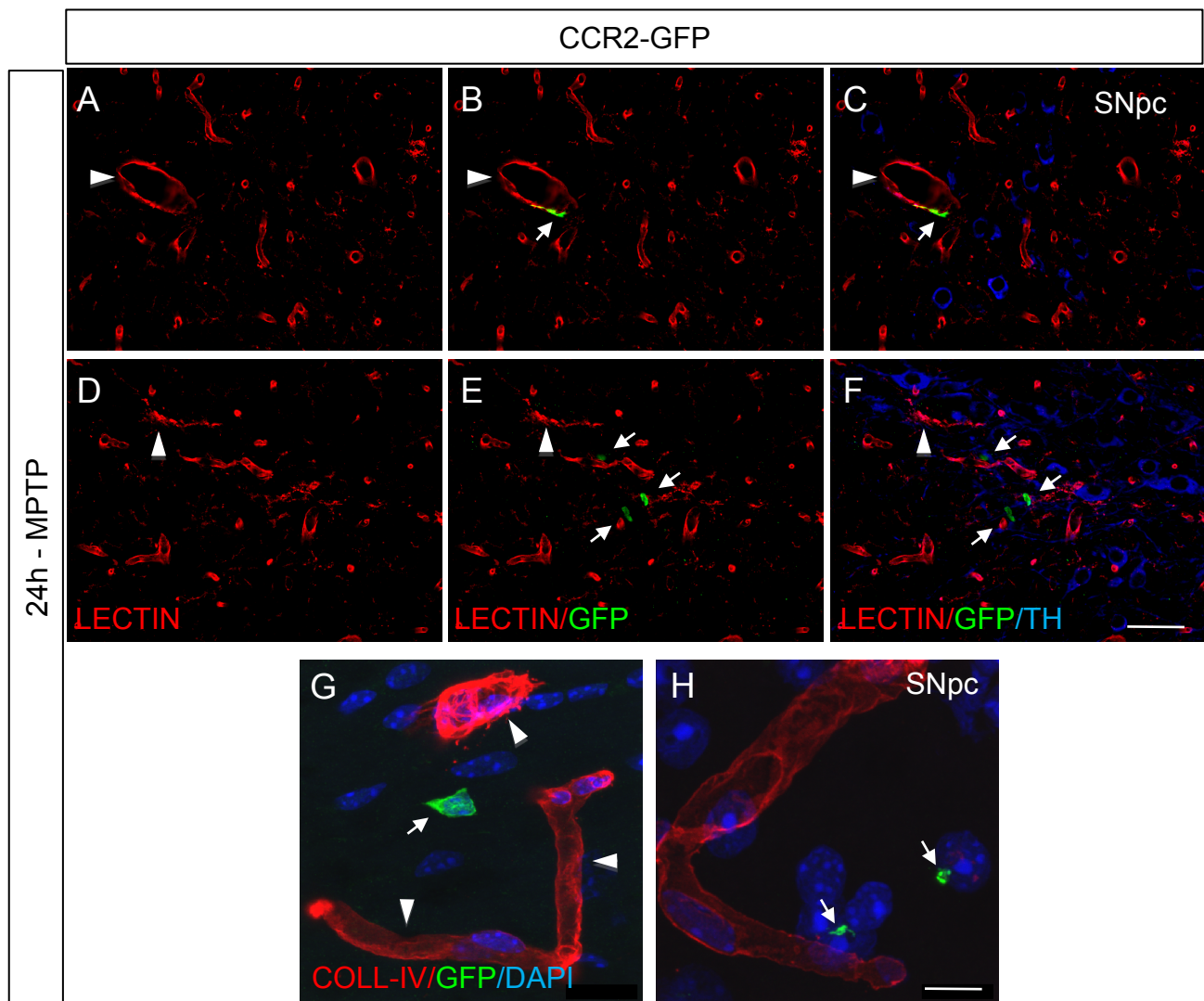

Suppl. Figure 6.

Supplement: Additional file 7: Figure S6. — CCR2+ monocytes infiltrating the affected substantia nigra in MPTP mice are present both perivascular and parenchymal. (A-F) Immunofluorescence stainings within the SNpc (anti-TH, blue) of CCR2-GFP mice, 24 h after MPTP intoxication to show both (A-C) CCR2-GFP+ cells (anti-GFP, green, arrows) with a localization at blood vessel endothelial cells (lectin, red, arrowheads), as well as (D-F) with a localization within the parenchyma, consistent with recently infiltrated CCR2-GFP+ monocytes. Of note, lectin can also mark microglia, but at the concentration used, mostly vessels. (G-H) Confirmatory stainings to show a specific vessel marker (anti-Collagen-IV; red, arrowheads), with parenchymal localization of CCR2-GFP+ monocytes in the SNpc (GFP; green, arrows). (Scale bars: F, H, 50 μm). (PDF 3580 kb) [file 12974_2017_830_MOESM7_ESM.pdf]

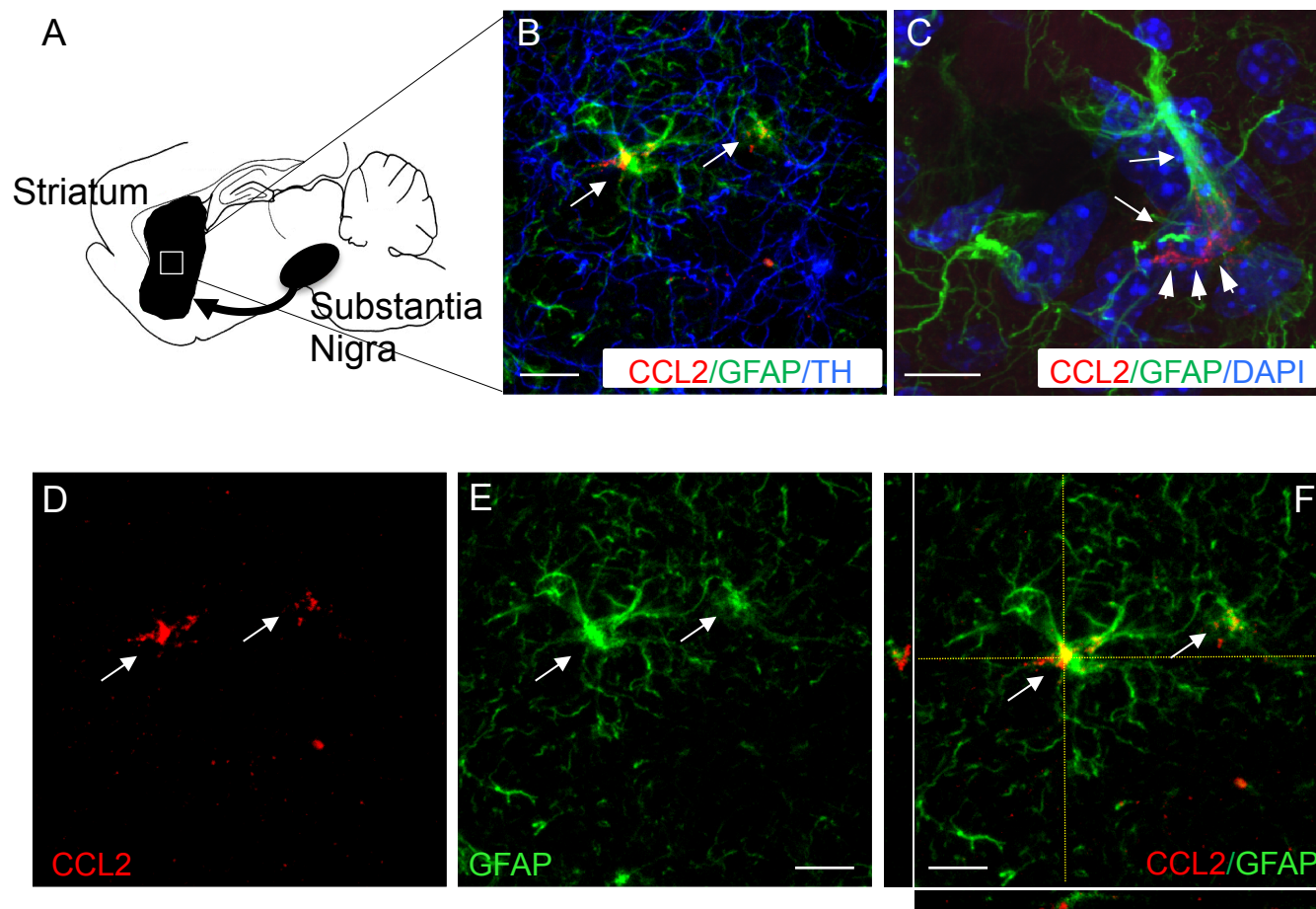

Suppl. Figure 7.

Supplement: Additional file 8: Figure S7. — Induction of CCL2 within astrocytes in the striatum of MPTP mice. (A) Schematic drawing of the nigro-striatal pathway of DA neurons. (B-F) Immunofluorescence stainings showing astrocytic localization (GFAP; green, arrow) of CCL2 induction (red, arrows) in the striatum (TH, DA innervation; blue) at 24 h after MPTP intoxication (no staining was detectable in saline injected mice, data not shown). (B) Example of a cytoplasmic CCL2 staining (shown as a confocal analysis in D-F). (C) Rare example of a potential extracellular CCL2 staining (red, arrowheads; with DAPI as a nuclear marker, blue) in close proximity to astrocytic processes (GFAP; green, arrows); only detected in the striatum, not in the SNpc. Of note, CCL2 induction was less prominent in the striatum (including at later timepoints than 24 h, data not shown) than in the SNpc, (see Figs. 2 and 3). (Scale bars; B, C, F, 20 μm). (PDF 1646 kb) [file 12974_2017_830_MOESM8_ESM.pdf]

A

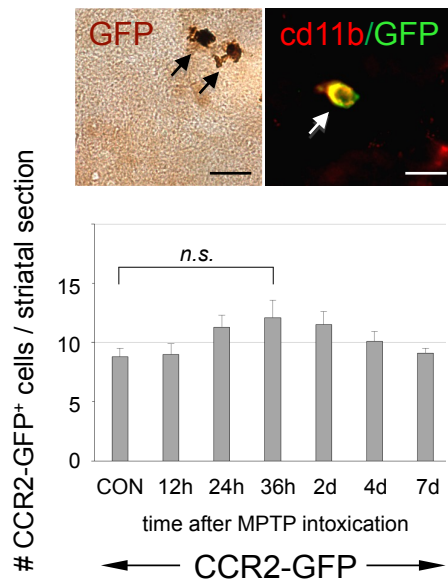

B

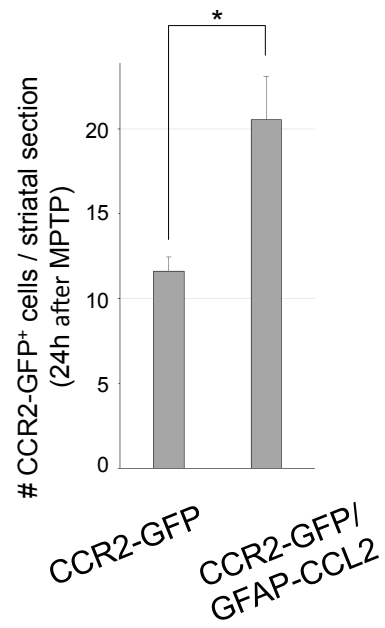

C

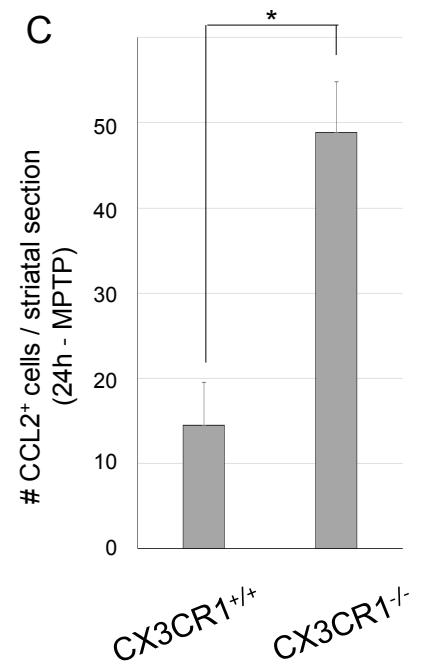

Suppl. Figure 8.

Supplement: Additional file 9: Figure S8. — Quantitative analysis of presence of CCR2+ monocytes and induction of CCL2 within the striatum of MPTP mice. (A) Full timecourse analysis of the presence of CCR2-GFP+ cells (inset left; GFP, brown) within the striatum of MPTP treated CCR2-GFP mice (from 12 h to 7 days after MPTP intoxication). CCR2-GFP+ cells (inset right) were double-positive for GFP (green) and cd11b (red), identifying them as monocytes. While there was a trend for an increase at 36 h after MPTP intoxication, this did not reach statistical significance (n.s.; P > 0.05; Kruskal-Wallis test). Of note, in contrast to the striatum, in the substantia nigra, an increased CCR2+ monocyte infiltration was measured at 36 h after intoxication (see, Fig. 4d). Counts represent average CCR2-GFP+ cells within a striatal section (means +/− SEM; 5–10 section counted; n = 4 mice per condition). (Scale bar; A, 10 μm). (B) Analysis of striatal CCR2+ monocyte infiltration in MPTP mice with transgenic over-induction of astrocytic CCL2. A significant increase in striatal CCR2+ monocyte infiltration was measured (at 24 h after MPTP intoxication) in CCR2-GFP/GFAP-CCL2 mice as compared to CCR2-GFP littermates (n = 4 mice per condition; *P < 0.05; t test). Of note, this was comparable to the significant increased CCR2+ monocyte infiltration that was detectable (at 24 h after intoxication) in the substantia nigra of CCR2-GFP/GFAP-CCL2 mice (see, Fig. 6c). (C) Analysis of striatal astrocytic CCL2 induction in MPTP mice with deletion for CX3CR1. A significant increased numbers of astrocytes inducing CCL2 was measured (at 24 h after intoxication) in CX3CR1−/− mice as compared to wild-type CX3CR1+/+ littermates (n = 4 mice per condition; *P < 0.05; t test). Of note, this was comparable to the significant increased numbers of astrocytes inducing CCL2 that was detectable in the substantia nigra of CX3CR1-deleted mice (see, Fig. 7e). (PDF 682 kb) [file 12974_2017_830_MOESM9_ESM.pdf]

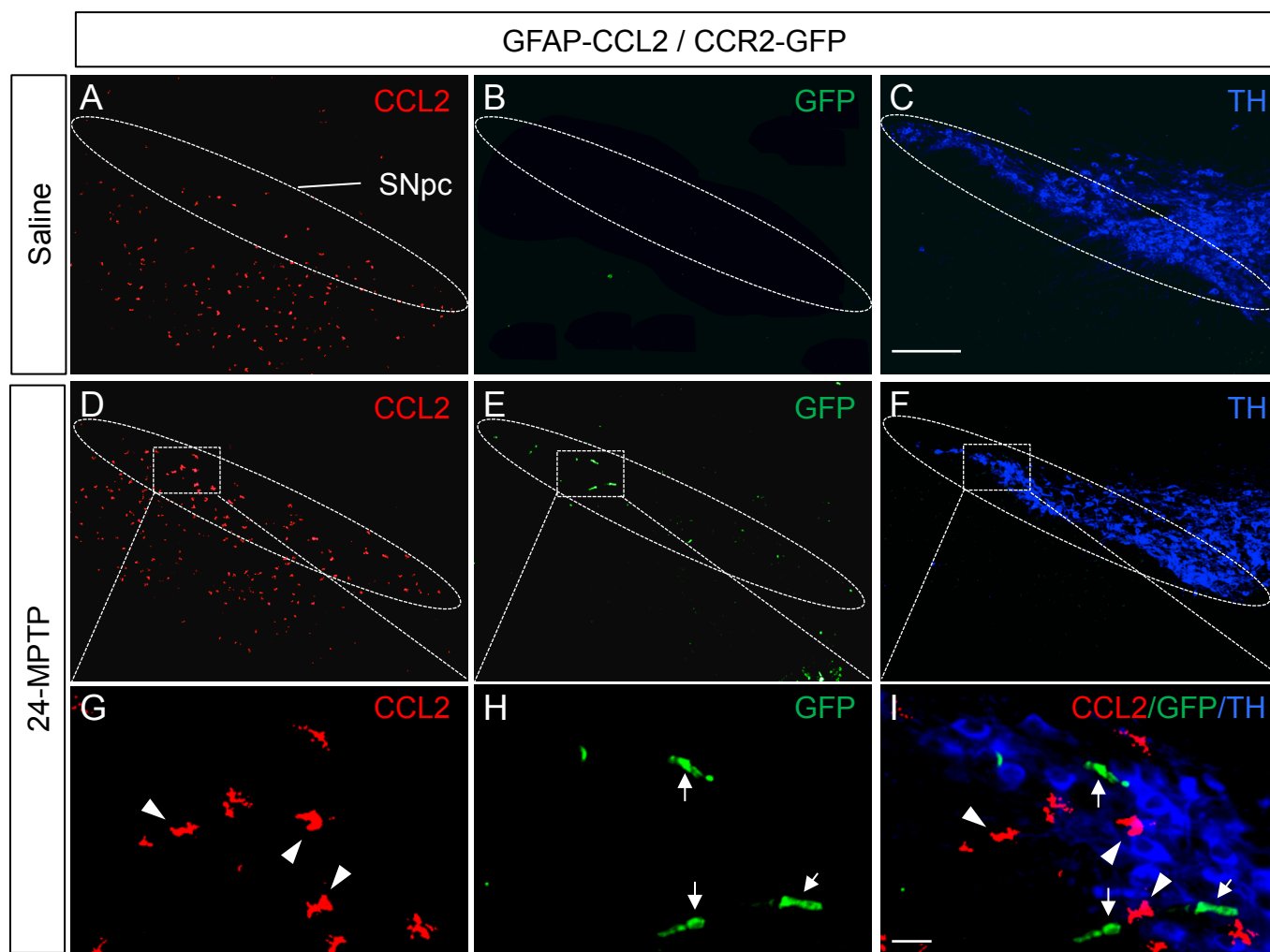

Suppl. Figure 9.

Supplement: Additional file 10: Figure S9. — Confirmation of CCL2 overexpression in GFAP-CCL2 mice. (A-C) Immunofluorescence stainings showing that baseline overexpression of CCL2 (red) in control (saline-injected) GFAP-CCL2/CCR2-GFP double-transgenic mice is mostly localized around, but less within the SNpc (A), which is marked with TH (blue). Only very rare CCR2-GFP+ cells (green) are detected under baseline conditions in or around the SNpc (B). (D-I) Strong CCL2 induction (red) is detected in the SNpc (marked with TH, blue) of MPTP treated GFAP-CCL2/CCR2-GFP double transgenic mice (D and inset, G), leading to increased presence of CCR2-GFP+ cells (green) in the affected SNpc (E-F and insets, H-I) (measured at 24 h after intoxication). (Scale bars; B, 200 μm; I, 20 μm). (PDF 1619 kb) [file 12974_2017_830_MOESM10_ESM.pdf]
