# Supplementary material for: Analysis of monocyte infiltration in MPTP mice reveals that microglial CX3CR1 protects against neurotoxic over-induction of monocyte-attracting CCL2 by astrocytes
Source: J Neuroinflammation. 2017 Mar 21;14:60. doi: 10.1186/s12974-017-0830-9 (PMC5359822; doi:10.1186/s12974-017-0830-9)
Supplement: Additional file 2: Table S1. — RNA profiling of chemokines in the laser-microdissected substantia nigra of the MPTP mouse model. Shown are results from the RT-qPCR profiling of 96 genes using TaqMan 384-well microfluidic cards, to assess all 61 chemokine family members (37 ligands, 24 receptors) as well as 32 other selected neuroinflammation-linked genes, normalized to Hprt1. RNA profiling was done in laser-microdissected SNpc at 2, 4, and 7 days after acute MPTP intoxication in adult 12 weeks old C57BL/6J males and compared to saline-injected controls (n = 9 mice per condition; as biological replicates). qPCR results are shown as normalized and averaged fold-changes (FC) to saline controls. In addition, the raw averaged Ct values of both the control and the 2 days (MPTP) samples are shown. Ct values of >30 are called absent (Abs). Only FC values that are statistically significant (P < 0.05; qBasePlus, Biogazelle) are indicated. Of note, the large FC values can be explained by both the enrichment provided by the LMD and the often very low expression in baseline control conditions. (nc, expressed but no significant change and N.D., non-determined) (* asterisks mark unclear corresponding receptors; Griffith et al. 2014). (PDF 163 kb) [file 12974_2017_830_MOESM2_ESM.pdf]

Table 1A: RNA profiling of Chemokines in the laser microdissected SNpc of MPTP mice.
(general neurodegenerative markers)

Gene Symbol		Ct-value Control	Ct-value 2 days	*days after MPTP (acute)* 2 days	4 days	7 days
				fold change (RNA/qPCR)		
**expression of dopaminergic and gliosis marker**						
**Th**		**13,3**	**14,9**	**-1,8**	**-1,5**	**-1,6**
**Gfap**		**18,3**	**13,2**	**59,2**	**51,5**	**85,1**
**Mhc-II**		**25,6**	**21,0**	**33,6**	**27,8**	**42,6**
**Cd68**		**21,7**	**17,7**	**27,2**	**33,4**	**41,0**

Table 1B: RNA profiling of Chemokines in the laser microdissected SNpc of MPTP mice.
(chemokine ligands and receptors)

Gene Symbol	Receptor	Ct-value Control	Ct-value 2 days	*days after MPTP (acute)* 2 days	4 days	7 days		Gene Symbol	Ct-value Control	Ct-value 2 days	*days after MPTP (acute)* 2 days	4 days	7 days
				fold change (RNA/qPCR)							*fold change (RNA/qPCR)*		
**Chemokine Ligand expression:**								**expression of the main corresponding Chemokine Receptors:**					
(Profiles correspond to Ligands only)													
Profile A - Early Peak Induction:													
**CCL2 (MCP-1)**	CCR2	**27,5**	**20,8**	**192,7**	**12,2**	**8,4**		**CCR2**	**29,4**	**24,8**	**37,7**	**15,8**	**12,9**
**CCL7 (MCP-3)**	CCR2/3	**28,3**	**24,7**	**29,6**	**5,2**	**3,1**		**CCR2**	**29,4**	**24,8**	**37,7**	**15,8**	**12,9**
**CCL12 (MCP-5)**	CCR2	**25,1**	**17,7**	**300,1**	**19,7**	**13,8**		**CCR2**	**29,4**	**24,8**	**37,7**	**15,8**	**12,9**
**CCL3 (MIP-1a)**	CCR5/1	**26,2**	**18,1**	**463,3**	**134,0**	**185,8**		**CCR5**	**24,4**	**23,3**	**3,6**	**4,9**	**4,6**
**CCL4 (MIP-1b)**	CCR5	**28,8**	**18,7**	**1419,3**	**181,4**	**176,9**		**CCR1**	**27,5**	**25,0**	**9,8**	**5,4**	**3,9**
**CXCL10 (IP-10)**	CXCR3	**28,3**	**16,6**	**4814,2**	**616,1**	**289,8**		**CXCR3**	**27,0**	**23,8**	**15,5**	**14,1**	**21,2**
**CXCL11 (I-TAC)**	CXCR3	**30,9**	**25,0**	**157,4**	**17,4**	**11,3**		**CXCR3**	**27,0**	**23,8**	**15,5**	**14,1**	**21,2**
**CCL8 (MCP-2)**	CCR1/2/3/5	**29,1**	**25,0**	**19,5**	**8,0**	**3,6**		**CCR1**	**27,5**	**25,0**	**9,8**	**5,4**	**3,9**
**CCL22 (MDC)**	CCR4	**28,5**	**27,4**	**3,7**	**1,7**	**1,9**		**CCR4**	*Abs*	*Abs*	*Abs*	*Abs*	*Abs*
**CXCL2 (GROb)**	CXCR2	**33,7**	**28,8**	**32,8**	**24,7**	**1,7**		**CXCR2**	**28,0**	**27,1**	**-7,9**	**-3,7**	**-5,6**
Profile B - Persistent Upregulation:													
**CXCL9 (MIG)**	CXCR3	*Abs*	**23,7**	**108,6**	**192,6**	**131,7**		**CXCR3**	**27,0**	**23,8**	**15,5**	**14,1**	**21,2**
**CXCL4 (Pf4)**	CXCR3 *	**26,0**	**25,3**	**2,8**	**2,8**	**3,1**		**CXCR3**	**27,0**	**23,8**	**15,5**	**14,1**	**21,2**
**CXCL16 (SR-PSOX)**	CXCR6	**24,6**	**19,0**	**76,2**	**44,4**	**59,6**		**CXCR6**	**31,1**	**26,1**	**35,4**	**30,0**	**58,0**
**CXCL14 (BRAK)**	*unknown*	**17,1**	**16,7**	**2,3**	**1,9**	**2,2**		--	--	--	--	--	--
**CCL9 (MIP-1g)**	CCR1 *	**24,3**	**20,7**	**19,7**	**16,6**	**26,1**		**CCR1**	**27,5**	**25,0**	**9,8**	**5,4**	**3,9**
**CCL5 (RANTES)**	CCR5/3/1	**27,6**	**20,9**	**162,9**	**165,0**	**228,2**		**CCR5**	**24,4**	**23,3**	**3,6**	**4,9**	**4,6**
**CCL11 (EOTAXIN)**	CCR3	**26,4**	**25,6**	**2,8**	**2,1**	**2,0**		**CCR3**	*Abs*	*Abs*	*Abs*	*Abs*	*Abs*
**CCL19 (MIP-3b)**	CCR7	**25,4**	**25,2**	**2,0**	**1,8**	**2,1**		**CCR7**	*Abs*	*Abs*	*Abs*	*Abs*	*Abs*
**CXCL5/6 (ENA-78/GCP-2)**	CXCR2/1	**26,6**	**26,1**	**2,3**	**3,9**	**3,3**		**CXCR2**	**28,0**	**27,1**	**-7,9**	**-3,7**	**-5,6**
**CXCL1 (GROa)**	CXCR2	**29,2**	**28,6**	**2,4**	**2,4**	**2,3**		**CXCR2**	**28,0**	**27,1**	**-7,9**	**-3,7**	**-5,6**
Profile C - Late Upregulation:													
**CCL6 (MRP-1)**	CCR5 *	**23,3**	**20,4**	**13,1**	**21,2**	**40,2**		**CCR5**	**24,4**	**23,3**	**3,6**	**4,9**	**4,6**
**xCL1 (LYMPHOTACTIN)**	XCR1	**33,6**	**28,6**	**27,6**	**40,6**	**62,1**		**xCR1**	*Abs*	**29,4**	**26,9**	*nc*	**3,6**
Profile D – Downregulation:													
**CCL28 (MEC)**	CCR10/3	**23,8**	**25,4**	**-1,8**	**-1,5**	**-1,6**		**CCR10**	**28,0**	**28,6**	*nc*	*nc*	*nc*
**CCL20 (MIP-3a)**	CCR6	**25,4**	**27,0**	**-1,8**	**-2,1**	**-2,0**		**CCR6**	**29,8**	**29,5**	**2,6**	**5,5**	**9,2**
**CCL17 (TARC)**	CCR4	**28,9**	**29,0**	*nc*	*nc*	**-2,1**		**CCR4**	*Abs*	*Abs*	*Abs*	*Abs*	*Abs*
**CXCL13 (BCA-1)**	CXCR5	**23,9**	**24,8**	*nc*	*nc*	**-1,9**		**CXCR5**	*Abs*	**33,0**	**12,5**	*nc*	**8,0**
mixed regulation:													
**CCL24 (EOTAXIN-2)**	CCR3	**27,4**	**28,8**	**-1,6**	**1,5**	**1,7**		**CCR3**	*Abs*	*Abs*	*Abs*	*Abs*	*Abs*
not changed:													
**CCL21 (TCA-4)**	CCR7/6	**26,2**	**26,8**	*nc*	*nc*	*nc*		**CCR7**	*Abs*	*Abs*	*Abs*	*Abs*	*Abs*
**CCL25 (TECK)**	CCR9	**21,0**	**21,5**	*nc*	*nc*	*nc*		**CCR9**	**27,4**	**27,5**	**1,5**	**1,5**	**1,6**
**CCL27a (ESKINE)**	CCR10	**18,1**	**18,9**	*nc*	*nc*	*nc*		**CCR10**	**28,0**	**28,6**	*nc*	*nc*	**-1,3**
**CXCL3 (GROg)**	CXCR2	**29,9**	**29,8**	*nc*	*nc*	*nc*		**CXCR2**	**28,0**	**27,1**	**-7,9**	**-3,7**	**-5,6**
**CXCL7 (PPBP/NAP-2)**	CXCR2	**24,6**	**25,1**	*nc*	*nc*	*nc*		**CXCR2**	**28,0**	**27,1**	**-7,9**	**-3,7**	**-5,6**
**CXCL12 (SDF-1)**	CXCR4	**21,8**	**22,2**	*nc*	*nc*	*nc*		**CXCR4**	**24,9**	**24,4**	**2,3**	**5,2**	**9,6**
**CX3CL1 (FRAKTALKINE)**	CX3CR1	**16,5**	**17,1**	*nc*	*nc*	*nc*		**CX3CR1**	**18,9**	**17,2**	**5,8**	**6,1**	**9,5**
**CCL1 (TCA-3)**	CCR8	*Abs*	*Abs*	*Abs*	*Abs*	*Abs*		**CCR8**	*Abs*	**32,5**	**6,7**	*nc*	**28,0**
**CCL26 (EOTAXIN-3)**	CCR3	*Abs*	*Abs*	*Abs*	*Abs*	*Abs*		**CCR3**	*Abs*	*Abs*	*Abs*	*Abs*	*Abs*
**CXCL17**	CXCR8	*Abs*	*Abs*	*Abs*	*Abs*	*Abs*		**CXCR8**	*ND*	*ND*	*ND*	*ND*	*ND*
								**Decoy Chemokine Receptors**					
								**DARC**	**23,6**	**23,5**	**1,9**	**1,7**	*nc*
								**D6**	**27,4**	**27,5**	*nc*	**1,6**	*nc*
								**CCX-CKR**	*Abs*	*Abs*	*Abs*	*Abs*	*Abs*

Table 1C: RNA profiling of Chemokines in the laser microdissected SNpc of MPTP mice.
(additional inflammation related genes implicated in neuro-glia-immune interactions)

				days after MPTP (acute)		
Gene Symbol		Ct-value	Ct-value	**2 days**	**4 days**	**7 days**
		Control	2 days	*fold change (RNA/qPCR)*		
**expression of general inflammatory genes**						
**Markers of glial reactivity**						
**B2m**		**17,8**	**15,9**	**6,0**	**8,1**	**8,4**
**Cd11c**		**29,3**	**23,6**	**83,9**	**103,8**	**728,6**
**Cd68**		**21,7**	**17,7**	**27,2**	**33,4**	**41,0**
**Gfap**		**18,3**	**13,2**	**59,2**	**51,5**	**85,1**
**Lilrb3**		**27,1**	**24,8**	**8,8**	**11,4**	**10,6**
**Mhc-II**		**25,6**	**21,0**	**33,6**	**27,8**	**42,6**
**Lymphocyte markers**						
**Cd3e**		**32,3**	**26,6**	**60,9**	**58,1**	**112,9**
**Foxp3**		*Abs*	*Abs*	*Abs*	*Abs*	*Abs*
**Il17a**		*Abs*	*Abs*	*Abs*	*Abs*	*Abs*
**Rorc**		**23,6**	**23,9**	*nc*	**3,8**	**5,3**
**Tbx21**		**20,2**	**19,2**	**22,0**	**28,4**	**38,4**
**Inflammatory/adhesion molecules**						
**Il1b**		**30,1**	**29,3**	**1,8**	**2,0**	**2,5**
**Icam1**		**27,5**	**24,4**	**14,9**	**9,0**	**15,5**
**Itgae**		**22,7**	**23,9**	*nc*	**-1,6**	**-2,3**
**Itgb2**		**22,7**	**19,2**	**19,0**	**19,7**	**28,6**
**Madcam1**		**30,6**	**28,4**	**7,5**	**17,4**	**102,4**
**Selp**		*Abs*	*Abs*	*nc*	*nc*	*nc*
**Selplg**		**18,9**	**17,4**	**4,8**	**5,7**	**5,9**
**Timp1**		**29,7**	**19,2**	**2518,0**	**225,8**	**215,7**
**Vcam1**		**20,2**	**19,2**	**3,4**	**3,1**	**3,3**
**Potential neuronal stress markers**						
**Cd200**		**17,0**	**18,0**	*nc*	*nc*	*nc*
**Cd200R1**		**27,5**	**25,1**	**9,2**	**8,8**	**16,4**
**Cd22**		**28,4**	**24,0**	**32,8**	**103,6**	**196,6**
**Cd44**		**25,2**	**20,1**	**70,9**	**22,1**	**26,8**
**Cd47**		**16,2**	**17,0**	*nc*	*nc*	*nc*
**Hspd1**		**20,5**	**21,1**	*nc*	*nc*	*nc*
**Mhc-I**		**20,0**	**17,5**	**9,2**	**13,2**	**13,5**
**Mmp3**		*Abs*	**28,5**	**12830,5**	**13520,5**	**181,5**
**NT-3**		**21,6**	**22,9**	**-1,5**	**-1,6**	**-1,7**
**Snca**		**14,3**	**15,6**	**-1,5**	**-1,5**	**-1,5**
**Spp1**		**19,7**	**18,6**	**3,7**	**2,6**	**8,7**
**Trem2**		**20,3**	**17,8**	**9,2**	**17,3**	**34,9**

Suppl. Table 1.
